# CDK4/CDK6 Inhibitors Synergize with Midostaurin, Avapritinib, and Nintedanib in Inducing Growth Inhibition in *KIT* D816V^+^ Neoplastic Mast Cells

**DOI:** 10.3390/cancers14133070

**Published:** 2022-06-23

**Authors:** Mathias Schneeweiss-Gleixner, Yüksel Filik, Gabriele Stefanzl, Daniela Berger, Irina Sadovnik, Karin Bauer, Dubravka Smiljkovic, Gregor Eisenwort, Nadine Witzeneder, Georg Greiner, Gregor Hoermann, Ana-Iris Schiefer, Juliana Schwaab, Mohamad Jawhar, Andreas Reiter, Wolfgang R. Sperr, Michel Arock, Peter Valent, Karoline V. Gleixner

**Affiliations:** 1 Ludwig Boltzmann Institute for Hematology and Oncology, Medical University of Vienna, 1090 Vienna, Austria; mathias.schneeweiss@meduniwien.ac.at (M.S.-G.); yueksel.filik@onc.lbg.ac.at (Y.F.); irina.sadovnik@meduniwien.ac.at (I.S.); bauer.karin@meduniwien.ac.at (K.B.); dubravka.smiljkovic@meduniwien.ac.at (D.S.); gregor.eisenwort@meduniwien.ac.at (G.E.); nadine.witzeneder@meduniwien.ac.at (N.W.); georg.greiner@meduniwien.ac.at (G.G.); gregor.hoermann@mll.com (G.H.); wolfgang.r.sperr@meduniwien.ac.at (W.R.S.); peter.valent@meduniwien.ac.at (P.V.); 2 Department of Internal Medicine I, Division of Hematology and Hemostaseology, Medical University of Vienna, 1090 Vienna, Austria; gabriele.stefanzl@meduniwien.ac.at (G.S.); daniela.berger@meduniwien.ac.at (D.B.); 3 Department of Medicine III, Division of Gastroenterology and Hepatology, Medical University of Vienna, 1090 Vienna, Austria; 4 Department of Laboratory Medicine, Medical University of Vienna, 1090 Vienna, Austria; 5 Ihr Labor, Medical Diagnostic Laboratories Vienna, 1220 Vienna, Austria; 6 MLL Munich Leukemia Laboratory, 81377 Munich, Germany; 7 Department of Pathology, Medical University of Vienna, 1090 Vienna, Austria; ana-iris.schiefer@meduniwien.ac.at; 8 Department of Hematology and Oncology, University Medical Center Mannheim and Medical Faculty Mannheim, Heidelberg University, 69120 Heidelberg, Germany; juliana.schwaab@medma.uni-heidelberg.de (J.S.); mohamad.jawhar@umm.de (M.J.); andreas.reiter@umm.de (A.R.); 9 Department of Hematological Biology, Pitié-Salpêtrière Hospital, Pierre et Marie Curie University (UPMC), 75013 Paris, France; michel.arock@lbpa.ens-cachan.fr

**Keywords:** systemic mastocytosis, *KIT* D816V, midostaurin, avapritinib, CDK4/CDK6, palbociclib, ribociclib, abemaciclib, targeted therapy, drug-combinations

## Abstract

**Simple Summary:**

Advanced systemic mastocytosis (AdvSM) is a rare malignant disease with a poor prognosis due to the drug resistance of neoplastic mast cells. We found that drugs targeting the cell cycle regulators CDK4 and CDK6 profoundly suppress the growth and survival of neoplastic mast cells. Furthermore, these drugs can overcome resistance against KIT D816V-targeting drugs, including midostaurin, in neoplastic mast cells. Finally, the CDK4/CDK6 inhibitors applied induced apoptosis in CD34+/CD38− stem cells in AdvSM. Based on these results, we believe that CDK4/CDK6 inhibition may be a new and interesting therapeutic approach with curative potential for AdvSM. Whether combinations of KIT D816-targeting drugs and CDK4/CDK6 inhibitors can induce long-term remission in patients with AdvSM remains to be determined in clinical trials.

**Abstract:**

In most patients with advanced systemic mastocytosis (AdvSM), neoplastic mast cells (MC) express *KIT* D816V. However, despite their disease-modifying potential, KIT D816V-targeting drugs, including midostaurin and avapritinib, may not produce long-term remissions in all patients. Cyclin-dependent kinase (CDK) 4 and CDK6 are promising targets in oncology. We found that shRNA-mediated knockdown of CDK4 and CDK6 results in growth arrest in the *KIT* D816V^+^ MC line HMC-1.2. The CDK4/CDK6 inhibitors palbociclib, ribociclib, and abemaciclib suppressed the proliferation in primary neoplastic MC as well as in all HMC-1 and ROSA cell subclones that were examined. Abemaciclib was also found to block growth in the drug-resistant MC line MCPV-1, whereas no effects were seen with palbociclib and ribociclib. Anti-proliferative drug effects on MC were accompanied by cell cycle arrest. Furthermore, CDK4/CDK6 inhibitors were found to synergize with the KIT-targeting drugs midostaurin, avapritinib, and nintedanib in inducing growth inhibition and apoptosis in neoplastic MCs. Finally, we found that CDK4/CDK6 inhibitors induce apoptosis in CD34+/CD38− stem cells in AdvSM. Together, CDK4/CDK6 inhibition is a potent approach to suppress the growth of neoplastic cells in AdvSM. Whether CDK4/CDK6 inhibitors can improve clinical outcomes in patients with AdvSM remains to be determined in clinical trials.

## 1. Introduction

Systemic mastocytosis (SM) is a rare disease characterized by the accumulation of neoplastic mast cells (MC) in one or more organs, such as the bone marrow (BM), the skin, the spleen, and the gastrointestinal tract [1,2,3,4]. SM can essentially be divided into indolent SM (ISM) and advanced SM (AdvSM) variants [1,2,3,4,5,6]. Although patients with ISM often suffer from mediator-related symptoms, their prognosis is excellent, with a normal or near-normal life expectancy [1,2,3,4,5,6]. In contrast, patients with AdvSM, including aggressive SM (ASM), MC leukemia (MCL), and SM with an associated hematologic neoplasm (SM-AHN), have a poor to very poor prognosis due to the drug resistance of neoplastic cells [3,4,5,6,7,8]. In these patients, organ damage induced by infiltrating neoplastic MC (C-Findings) and disease progression are seen, resulting in reduced survival [3,4,5,6,7,8]. In most patients, the D816V-mutated variant of *KIT* is detected in neoplastic MC and is considered to act as a driver of disease evolution and MC expansion [9,10,11].

Several pharmacologic approaches have been established to counteract the expansion of neoplastic MC in AdvSM, including cytostatic drugs, interferon-alpha, cladribine, poly-chemotherapy, allogeneic hematopoietic stem cell transplantation (HSCT), and KIT-targeting drugs [4,5,6,7,12,13,14,15,16,17,18,19,20,21]. Since KIT has been identified as a major target of therapy, research efforts have primarily focused on the identification of potent tyrosine kinase inhibitors (TKIs) blocking KIT D816V [13,14,16,17,18,19,20,21,22]. These efforts resulted in the approval of the KIT-targeting drug midostaurin for treatment of patients with AdvSM [23]. Indeed, midostaurin shows beneficial effects in a majority of patients with ASM and MCL [18,19,23,24]. However, responses are often transient and, in most patients, long-term responses and complete remissions are not obtained [16,18]. These observations suggest that midostaurin, when used as a single drug, may not be sufficient to achieve optimal disease control in AdvSM. Therefore, more potent TKIs directed against KIT D816V have been developed and tested in preclinical and clinical studies. One of these drugs is avapritinib, which has recently been approved by the FDA for application in patients with AdvSM, based on the superior effects of this drug [19,20,21,25,26,27,28,29]. However, not all patients with AdvSM respond to avapritinib and relapses, often in form of a *KIT* D816V-negative AHN or MCL, have been reported [25,28,29].

All in all, single agent KIT-targeting treatment may not be sufficient to suppress all cells and sub-clones in a complex disease such as AdvSM. Therefore, research focuses on identifying drug combinations that are able to induce more durable remissions in AdvSM. Indeed, a number of potential combination partners for midostaurin or other KIT D816V-targeting TKIs have been examined [14,30,31,32]. Recent data have shown that neoplastic cells in AdvSM exhibit somatic mutations in diverse additional driver genes apart from *KIT* [11,17,33,34,35,36,37,38]. These additional lesions and the related signaling pathways may support cell cycle progression and the expansion of neoplastic MC in AdvSM.

Cyclin-dependent protein kinases (CDKs) have various functions in normal and malignant cells. CDK4 and CDK6 regulate the activity of the retinoblastoma protein (RB1) and are required for G1 phase progression and G1/S transition of the cell cycle [39,40,41,42,43]. In the recent past, CDK4 and CDK6 have been established as major drug targets in applied oncology [39,40,41,42,43,44,45,46,47,48,49]. To date, three CDK4/CDK6 inhibitors, palbociclib, ribociclib, and abemaciclib, have been applied in patients with breast cancer [44,45,46,47,48,49]. All three inhibitors are relatively well-tolerated drugs [44,45,46,47,48]. Of note, abemaciclib, which is the most potent of the three inhibitors, is less selective than palbociclib and ribociclib and thus blocks a number of targets other than CDK4/CDK6 [39,49,50]. More recent pre-clinical studies have shown promising effects of CDK4/CDK6 inhibitors in certain hematologic malignancies [51,52,53,54]. However, the anti-neoplastic effects of drugs targeting CDK4/CDK6 have not previously been analyzed in the context of AdvSM.

In the current study, we explored the expression and role of CDK4/CDK6 in neoplastic MC and evaluated the anti-neoplastic effects of CDK4/CDK6-targeting drugs alone or in combination with KIT D816V-targeting TKI.

## 2. Methods

### 2.1. Reagents

Reagents used in this study are described in the Supplementary Materials.

### 2.2. Isolation of Primary Neoplastic Cells

Primary neoplastic cells were isolated from 21 BM samples of 17 patients with SM. In these 21 samples, the disease was classified as ISM (*n* = 6), ASM (*n* = 2), SM-AHN (*n* = 9), and MCL (*n* = 4), according to WHO criteria (Table 1) [3,7]. In three patients, samples were collected at different time points: one patient (#4) progressed from ISM to ASM and later to MCL, one patient (#3) progressed from ISM to ISM-AML, and one patient (#14) received treatment with avapritinib between the two timepoints of sampling (Table 1). BM cells were obtained during routine diagnostic investigations after written informed consent was obtained. BM samples were layered over Ficoll (density: 1.077 g/mL) to isolate mononuclear cells (MNC) as described in [54,55]. The percentage of neoplastic cells (MC or clonal cells in case of SM-AHN) in BM-smears ranged from 1–40% as determined by microscopy. Furthermore, RNA was obtained from the BM of 50 additional patients with SM classified as ISM (*n* = 27), SSM (*n* = 1), ASM (*n* = 5), SM-AHN (*n* = 11), and MCL (*n* = 6) (see Supplemental Materials, Table S1). The study was approved by the ethics committee of the Medical University of Vienna.

### 2.3. Culture of Human Cell Lines

The following human MCL-like cell lines were employed in this study: HMC-1.1 and HMC-1.2 [56], two ROSA sub-clones (ROSA^KIT WT^, ROSA^KIT D816V^) [55,57], and four MCPV-1 sub-clones (MCPV-1.1, MCPV-1.2, MCPV-1.3, MCPV-1.4) [58]. A detailed description of these cell lines is provided in the Supplementary Materials.

### 2.4. Quantitative Polymerase Chain Reaction and Western Blot Analysis

To determine the expression of *CDK4*, *CDK6*, *cyclin D1*, *cyclin D2* and *RB1* in BM samples obtained from patients with SM and in the MCL-related cell lines, RNA-isolation and a quantitative polymerase chain reaction (qPCR) were performed as reported in [54] using specific primers, as listed in the Supplemental Materials, Table S2. *ABL1* was used as reference gene. A detailed description of the technique is provided in the Supplementary Materials.

Expression of CDK4, CDK6, cyclin D1, cyclin D2, and RB1 protein was analyzed in MC cell lines by western blotting as described [54,59]. To study the effects of CDK4/CDK6 inhibitors on the primary target (RB1), HMC-1 cells were kept in control medium or in the presence of CDK4/CDK6 inhibitors (1 µM each) for 4 h. Thereafter, western blotting was performed essentially as described, using antibodies directed against RB1 and its phosphorylated variant (pRB1) as well as actin (loading control) [54,59]. In a different set of experiments, CDK4/CDK6 inhibitors (1 µM each) were applied in HMC-1 cells alone or in combination with 1 µM bortezomib for 4 h prior to western blot analysis. Induction of cleaved caspase-3 was analyzed to evaluate apoptosis in drug-exposed cells. ß-tubulin was used as the loading control. A list of antibodies used in the western blot experiments is provided in the Supplementary Materials, Table S3. All original blots as well as densitometric analysis are provided in the Supplementary Materials, Additional Information.

### 2.5. Evaluation of Drug Effects on Cell Proliferation

Primary neoplastic cells and cell lines were incubated in a control medium or various concentrations of CDK4/CDK6 inhibitors (palbociclib, ribociclib and abemaciclib applied at 0.005–10 µM) at 37 °C for 48 h. Thereafter, ^3^H-thymidine uptake was measured as previously described [54,55]. In a separate set of experiments, HMC-1 and ROSA cells were exposed to various concentrations of the CDK4/CDK6 inhibitors and KIT D816V-targeting drugs (midostaurin, avapritinib, nintedanib), either as single agents or in combination at a fixed ratio of drug concentrations before ^3^H-thymidine uptake was measured. All experiments were performed in triplicates. A detailed description of the technique is provided in the Supplementary Materials.

### 2.6. Evaluation of Apoptosis in Drug-Exposed Cells

For flow cytometric determination of apoptosis, cell lines were cultured in a control medium or in a medium supplemented with a CDK4/CDK6 inhibitor (0.5–10 μM), midostaurin (0.1–0.5 µM) or avapritinib (0.3–0.7 µM), or a combination of two drugs (‘CDK4/CDK6 inhibitor + midostaurin’ or ‘CDK4/CDK6 inhibitor + avapritinib’) at 37 °C for 48 or 72 h and subjected to combined Annexin V/4′,6-diamidino-2-phenylindole (DAPI) staining to determine apoptosis by flow cytometry as described [54,55]. In a separate set of experiments, primary BM MNC obtained from four patients with AdvSM were exposed to palbociclib, ribociclib or abemaciclib (1, 5 and 10 µM each) before apoptosis in CD34+/CD38− neoplastic stem cells was quantified by flow cytometry on a FACSCanto II (BD Biosciences, Franklin Lakes, NJ, USA) as described [60].

### 2.7. Evaluation of Drug-Induced Cell Cycle Arrest

For analysis of cell cycle progression, cell lines (HMC-1 and ROSA cells) were kept in the presence or absence of various concentrations of palbociclib, ribociclib, or abemaciclib for 24 h. Thereafter, propidium iodide was added and cell cycle distribution was analyzed as described previously [54]. These flow cytometric measurements were performed on a FACSCalibur (BD Biosciences).

### 2.8. Measurement of Histamine Release

Dextran-enriched blood basophils obtained from healthy individuals (*n* = 3) were incubated in a control medium or in the presence of palbociclib, ribociclib, or abemaciclib (0.01–10 μM each) at 37 °C for 30 min. Then, histamine release was determined as described [55]. A detailed description of the technique is provided in the Supplementary Materials.

### 2.9. shRNA-Mediated Knockdown of CDK4 and CDK6

HMC-1.1 and HMC-1.2 cells were transfected simultaneously with short hairpin RNA (shRNA) constructs directed against CDK4 (inducible knockdown with doxycycline; induced shRNA labeled with dsRed) and CDK6 (induced shRNA labeled with GFP) or with control shRNAs, as previously described (see also the Supplementary Materials and Supplemental Table S4) [54]. Knockdown of CDK4 and CDK6 was confirmed by western blotting in puromycin-selected, doxycycline-induced cells. After transfection and incubation with doxycycline, transduced cells were sorted for dsRed or GFP positivity on a BD FACSAria Fusion (Becton Dickinson, Franklin Lakes, NJ, USA). HMC-1.2 cells transfected with two non-targeting control shRNAs were also run through the sorter to provide equal conditions. Thereafter, HMC-1.2 cells transfected with either targeting or non-targeting shRNAs were mixed with un-transduced HMC-1.2 cells at a 1:1 ratio and were cultured for 10 days. The percentage of GFP+/dsRed+ cells was monitored by flow cytometry on days 1, 3, 5, and 10. A detailed description of shRNA-mediated knockdown is provided in the Supplementary Materials.

### 2.10. Statistical Analysis

To determine the level of significance of differences seen in growth inhibition, cell cycle progression, and apoptosis studies, analysis of variance testing (ANOVA) with post-testing using the Dunnett test was applied. Results were considered to be significantly different when *p* was <0.05. In drug combination experiments, drug interaction-types were determined by calculating combination index (CI) values using Calcusyn software [61]. A CI of <1 indicates a synergistic effect. Furthermore, synergy scores (zero interaction potency model = ZIP) were determined by SynergyFinderPlus software [62,63]. A synergy score >10 stands for synergism, a synergy score between -10 and 10 indicates an additive effect, and a synergy score < −10 stands for antagonism. For western blot experiments, protein expression levels (determined by densitometric analysis) between two conditions were compared using the Student’s *t* test. The results were considered to be significantly different when *p* was <0.05.

## 3. Results

### 3.1. Identification of CDK4/CDK6 as Potential Therapeutic Targets in Neoplastic MC

**Figure 1:**
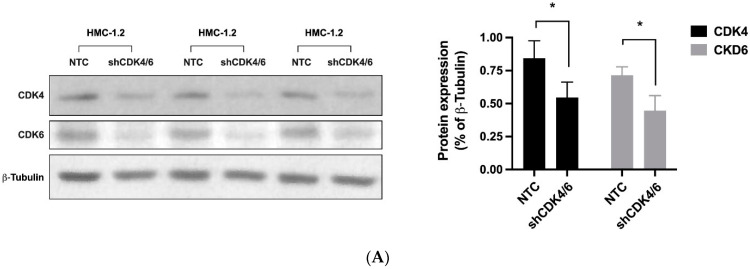

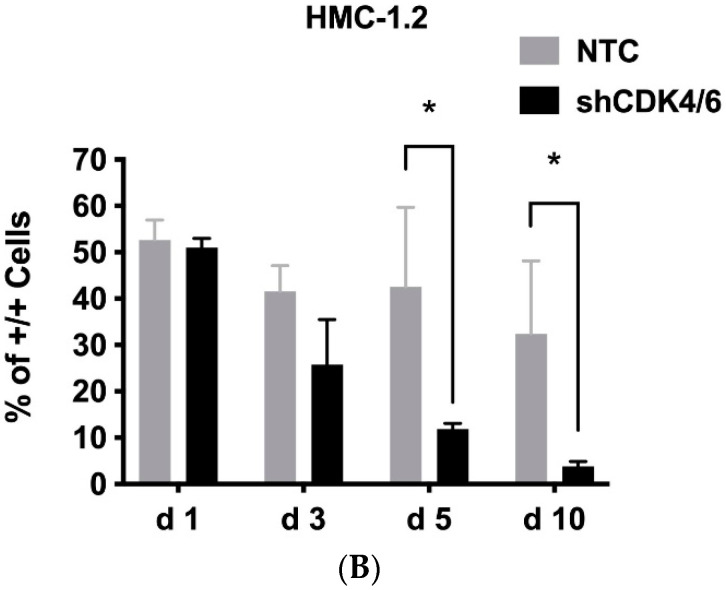
The CDK4/CDK6 pathway represents a therapeutic target in neoplastic MC. HMC-1.2 cells were simultaneously transfected with shRNA directed against CDK4 and CDK6 (shCDK4/6; both inducible with doxycycline) or with two non-targeting control shRNAs (NTC) labeled with GFP/RFP. (**A**): Transfected cells were selected by puromycin and induced with doxycycline for 72 h. Thereafter, knockdown of CDK4 and CDK6 was confirmed by western blotting in triplicate (left panel). Protein expression (as ratio of ß-tubulin) was determined by densitometric analysis. The mean protein expression was calculated for each condition (right panel). Asterisk (*): *p* < 0.05. (**B**): After 72 h of doxycycline exposure, GFP+/RFP+ cells were FACS-sorted. FACS-sorted HMC-1.2 cells transfected with two non-targeting control shRNAs (NTC) or with shRNA directed against CDK4 and CDK6 (shCDK4/6) were mixed with non-transfected HMC-1.2 cells at a 1:1 ratio and cultured for 10 days. The percentage of GFP+/RFP+ cells (+/+) was monitored by flow cytometry on days 1, 3, 5, and 10. Asterisk (*): *p* < 0.05. Abbreviations: CDK, cyclin-dependent kinase; shRNA, short hairpin RNA; GFP, green fluorescent protein; RFP, red fluorescent protein.

We first examined the expression of critical molecules involved in the CDK4/CDK6 pathway (CDK4, CDK6, cyclin D1, cyclin D2, RB1) in neoplastic MC. As evidenced by qPCR, transcripts of CDK4, CDK6, cyclin D1, cyclin D2, and RB1 were detectable in all BM samples analyzed (ISM: *n* = 27; SSM: *n* = 1; ASM: *n* = 5; SM-AHN: *n* = 11; MCL: *n* = 6). However, mRNA expression levels varied from patient to patient without a clear correlation to a subtype of SM (Supplemental Materials, Figure S1A). Neoplastic cells in AdvSM expressed slightly higher levels of CDK4, CDK6, cyclin D1, cyclin D2, and RB1 than the cells tested in non-advanced SM (Supplementary Materials, Figure S1B). However, these differences did not reach statistical significance. All five mRNA transcripts analyzed were also detected in the MCL-like cell lines tested (Supplementary Materials, Figure S1C). Interestingly, expression patterns differed among the cell lines: MCPV-1 cells expressed high levels of cyclin D1 but lower levels of cyclin D2 compared to HMC-1 and ROSA cells (Supplementary Materials, Figure S1C). Similar results were seen at the protein level: whereas CDK4, CDK6, cyclin D1 and RB1 were detected in all cell lines, cyclin D2 was not expressed in MCPV-1 cells as assessed by western blot analysis (Supplementary Materials, Table S5). In a next step, we analyzed the functional role of CDK4 and CDK6 in neoplastic MC. For this purpose, a simultaneous shRNA-mediated knockdown of CDK4 and CDK6 was performed in HMC-1.2 cells (Figure 1A). As evidenced by culturing a mix of transfected and untransfected HMC-1.2 cells, the simultaneous knockdown of CDK4 and CDK6 resulted in a significant growth disadvantage when compared to cells transduced with control shRNA (Figure 1B), suggesting that CDK4 and CDK6 play an important role in the proliferation of neoplastic MC and may, therefore, qualify as therapeutic targets.

### 3.2. Palbociclib, Ribociclib and Abemaciclib Disrupt CDK4/CDK6 Signaling in Neoplastic MC

**Figure 2:**
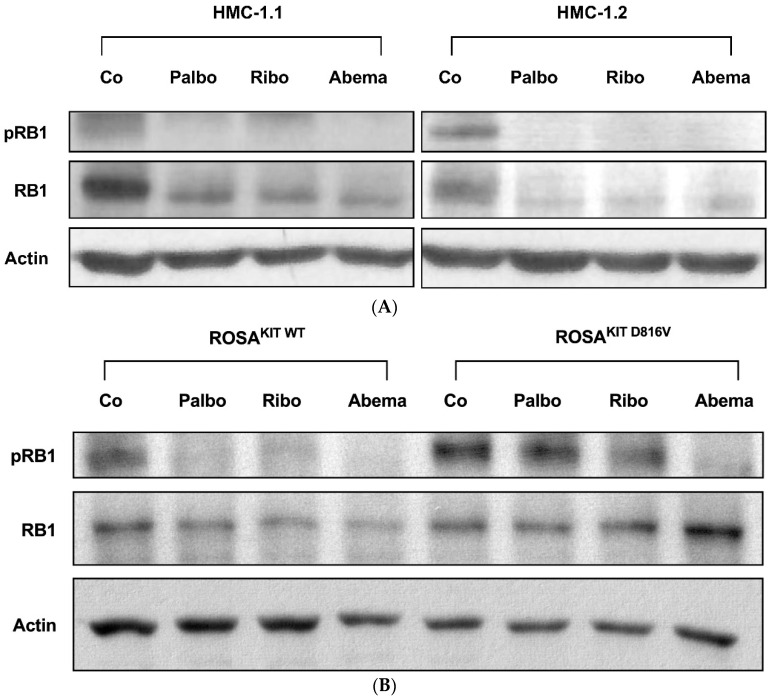

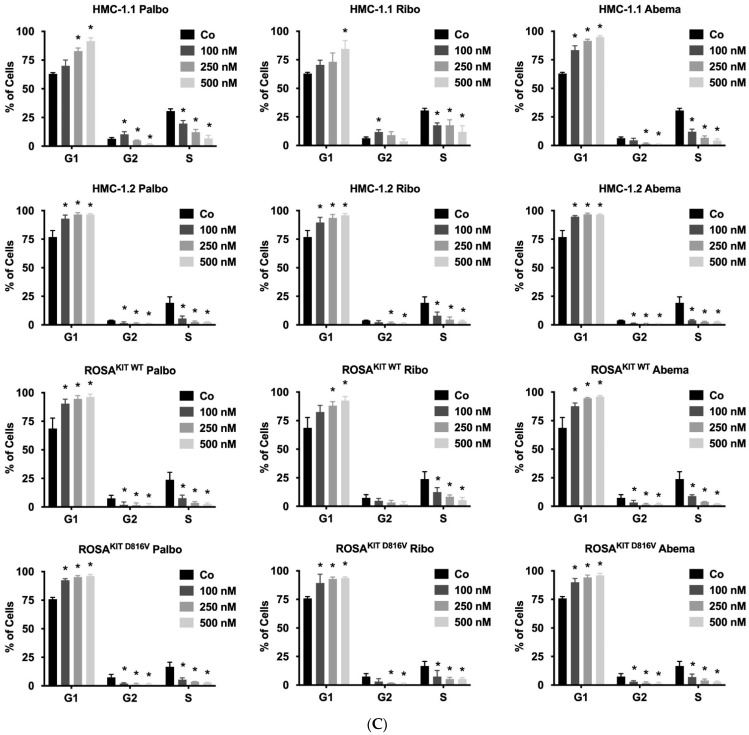

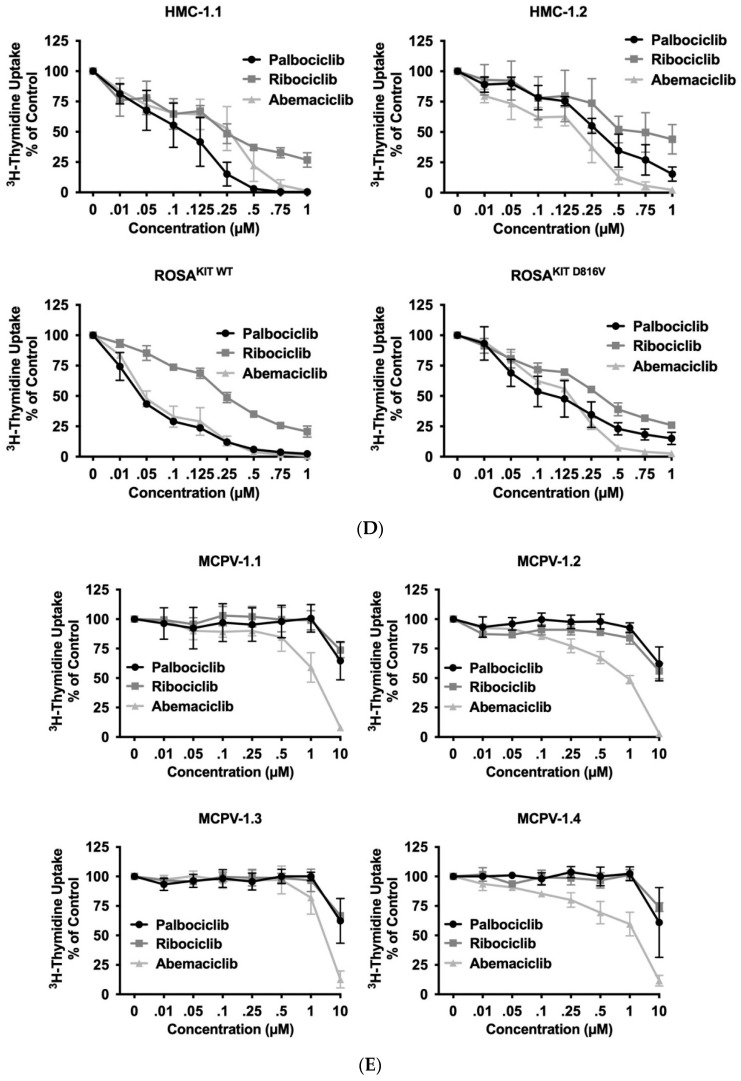

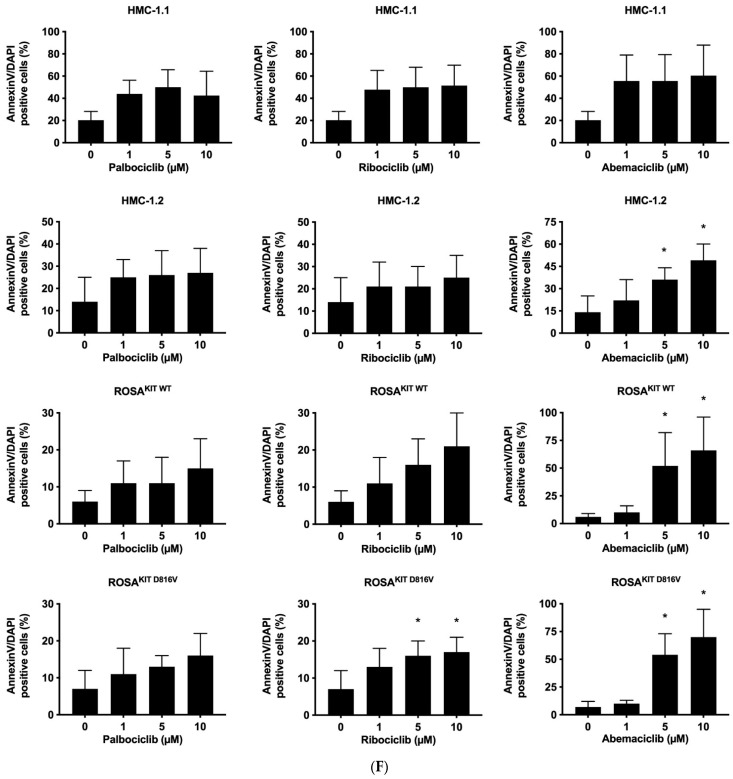

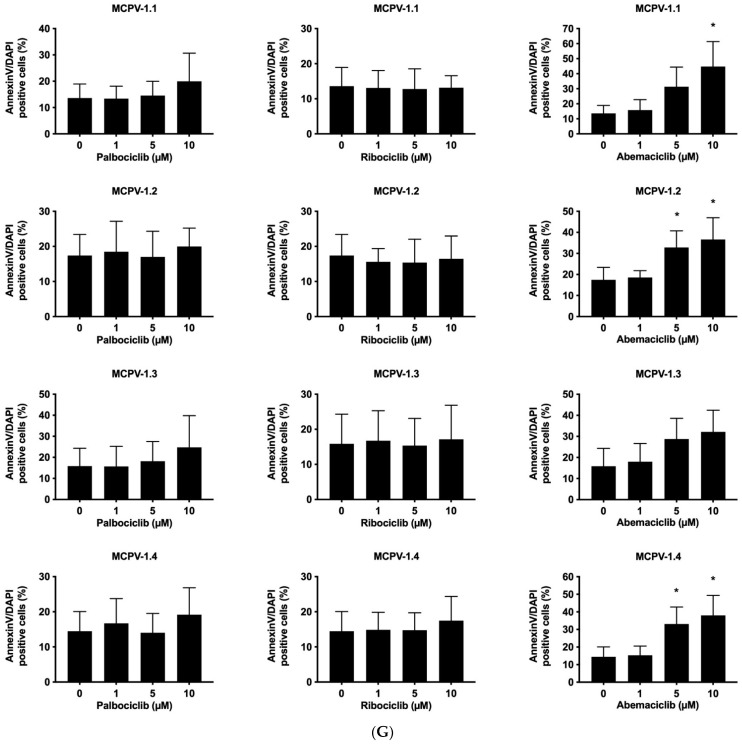
CDK4/CDK6 inhibitors counteract proliferation and survival of neoplastic mast cells (MCs) and induce cell cycle arrest by inhibiting phosphorylation of RB1. A–B: HMC-1 (**A**) and ROSA (**B**) cells were incubated in control medium (Co) or in medium containing 1 µM of palbociclib (Palbo), ribociclib (Ribo) or abemaciclib (Abema), as indicated, at 37 °C for 4 h. Thereafter, cells were harvested and western blotting was performed, as described in the text, using antibodies against phosphorylated RB1 (pRB1), total RB1, and actin. (**C**) HMC-1 and ROSA cells were kept in control medium (Co) or were incubated with different concentrations of palbociclib (Palbo), ribociclib (Ribo), or abemaciclib (Abema), as indicated, for 48 h. Thereafter, PI was added and cell cycle distribution was determined by flow cytometry. The percentage of cells in G1-phase, G2/M-phase, and S-phase in each condition are shown. To determine statistical significance, ANOVA and post-testing by Dunnett test were performed. Asterisk (*): *p* < 0.05. (**D**,**E**) HMC-1 and ROSA (**D**), as well as MCPV-1 cells (**E**), were incubated in control medium (0 nM) or medium containing various concentrations of palbociclib, ribociclib, or abemaciclib, as indicated, at 37 °C for 48 h. Thereafter, ^3^H-thymidine uptake was measured. Results are expressed as percentage of control and represent the mean ± S.D. from three independent experiments. Statistical significance was reached in all experiments, as analyzed by ANOVA and post-testing by Dunnett test (Supplementary Materials, Table S6). (**F**,**G**) HMC-1 and ROSA cells (**F**), as well as MCPV-1 cells (**G**), were incubated in control medium (0 μM) or medium containing various concentrations of palbociclib, ribociclib, or abemaciclib, as indicated, at 37 °C for 48 h. Thereafter, cells were harvested and AnnexinV/DAPI-positive cells were analyzed by flow cytometry. Results show the percentage of apoptotic cells and represent the mean ± S.D. of 4 independent experiments. To determine statistical significance, ANOVA and post-testing by Dunnett test were performed. Asterisk (*): *p* < 0.05. Abbreviations: RB1, retinoblastoma protein 1; pRB1, phosphorylated variant of RB1; nM, nanomolar; µM, micromolar.

Next, we were interested in the effects of pharmacologic CDK4/CDK6 inhibitors on neoplastic MC. In these experiments, we focused on three drugs that are currently applied in patients: palbociclib, ribociclib, and abemaciclib. In western blot experiments, all three compounds were found to downregulate the expression of pRB1 in HMC-1.1, HMC-1.2, ROSA^KIT WT^, and ROSA^KIT D816V^ cells (Figure 2A,B). Interestingly, in HMC-1 cells, not only phosphorylation, but also the expression of RB1, decreased after 4 h of incubation with the CDK4/CDK6 inhibitors, confirming previous observations in other malignant cell types [64,65,66]. To learn more about the mechanism underlying RB1 regulation, the expression of RB1 mRNA was analyzed by qPCR in HMC-1.1 and HMC-1.2 cells kept in control conditions or in the presence of palbociclib, ribociclib or abemaciclib (1 µM each) for 4 or 24 h. However, no significant differences were seen (not shown). We further exposed HMC-1.2 cells to a combination of 1 µM palbociclib + 1 µM bortezomib. Inhibition of the proteasome by bortezomib failed to counteract the effects of palbociclib on RB1 expression (not shown). Together, our findings suggest CDK4/CDK6 inhibitors can disrupt RB1-dependent downstream signaling in neoplastic MC. 

### 3.3. CDK4/CDK6 Inhibitors Counteract Proliferation and Survival in the Human MC Lines HMC-1 and ROSA Whereas Only Abemaciclib Is Effective in Drug-Resistant MCPV-1 Cells

In a next step, we investigated the anti-neoplastic effects of CDK4/CDK6 inhibitors in the MCL-related cell lines. As visible in Figure 2C, all three inhibitors induced cell cycle arrest in G1-phase in HMC-1 cells and ROSA cells expressing or lacking *KIT* D816V. In these cell lines, cell cycle arrest was accompanied by growth arrest, as assessed by ^3^H-thymidine uptake experiments, with IC_50_ values ranging between 23 nM and 703.8 nM (Table 2, Figure 2D). Confirming observations in other neoplastic cell types, abemaciclib was found to be a more potent inhibitor of the growth of neoplastic MC than palbociclib or ribociclib. In highly resistant MCPV-1 cells lacking *KIT* D816V but expressing *RAS* G12V, *Large T* and *hTert*, only abemaciclib induced growth inhibition and apoptosis at high drug concentrations (1–10 µM) (Table 2, Figure 2E). Growth inhibition reached statistical significance for all cell lines and all compounds tested (Supplementary Materials, Table S6). We next analyzed whether growth arrest would be accompanied by induction of apoptosis (Figure 2F,G; Supplementary Materials, Table S7). Indeed, an increase in the percentage of apoptotic cells was observed for all compounds tested in HMC-1 and ROSA cells, and for abemaciclib in MCPV-1 cells. However, the effects of CDK4/CDK6 inhibitors on the induction of apoptosis were less pronounced than their effects on cell cycle progression and cell proliferation. In fact, in most cell lines, statistical significance was only reached for abemaciclib when comparing the percentage of apoptotic cells. Overall, MCPV-1 cells were found to be largely resistant against the CDK4/CDK6 inhibitors applied, with the exception of abemaciclib when applied at higher doses. This observation may best be explained by the fact that abemaciclib, the least selective drug among the three approved CDK4/CDK6 inhibitors, may block additional targets in neoplastic cells [39,50].

### 3.4. CDK4/CDK6 Inhibitors Block the Proliferation of Primary Neoplastic Cells Isolated from Patients with Various Subtypes of SM including Relapsed MCL

**Figure 3:**
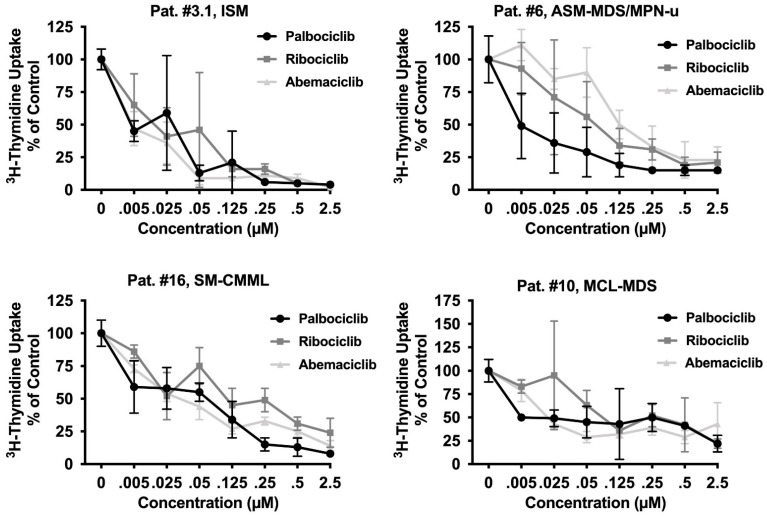
CDK4/CDK6 inhibitors induce growth arrest in primary neoplastic MC. Primary neoplastic MC obtained from one patient with ISM and three patients with AdvSM (as indicated) were incubated in control medium (0 nM) or medium containing various concentrations of palbociclib, ribociclib, or abemaciclib as indicated, at 37 °C for 48 h. Thereafter, ^3^H-thymidine uptake was measured. Results are expressed as percentage of control and represent the mean ± S.D from triplicates. Statistical significance was reached in all experiments, as analyzed by ANOVA and post-testing by Dunnett test (Supplementary Materials, Table S8). The patient’s number refers to Table 1. Abbreviations: SM, systemic mastocytosis; ISM, indolent SM; ASM, aggressive SM; MCL, mast cell leukemia, AML, acute myeloid leukemia; MDS, myelodysplastic syndrome; MPN, myeloproliferative neoplasm; CMML, chronic monomyelocytic leukemia; u, unspecified; µM, micromolar.

Together, our data reveal that pharmacologic inhibitors of CDK4/CDK6 exert strong anti-neoplastic effects in most MCL-like cell lines tested. To confirm these effects in primary neoplastic MC, 21 BM samples from a total of 17 patients with SM (Table 1) were tested. As visible in Table 1 and Figure 3, all three CDK4/CDK6 inhibitors induced growth arrest in primary cell samples. IC_50_-values ranged between 5 and 200 nM for palbociclib, 38 and 362 nM for ribociclib, and 5 and 160 nM for abemaciclib (Table 1). In none of the samples higher drug-doses (exceeding 1 µM) were needed to induce complete growth arrest. Growth inhibition reached statistical significance for all samples and all compounds tested (Supplementary Materials, Table S8). Thus, primary neoplastic MC were even more sensitive to CDK4/CDK6 inhibition than cell lines. Growth inhibition was observed in all subtypes of SM, including patients with *KIT* D816V^+^ ASM and MCL, and was independent of the presence or absence of an AHN. In the two patients who suffered from disease progression (from ISM to ASM/MCL or from ISM to ISM-AML), no loss of sensitivity against CDK4/CDK6 inhibitors was observed. Of note, in two patients with relapsed MCL following midostaurin treatment (patient #4.3 and #15; Table 1), all CDK4/CDK6 inhibitors tested also produced growth inhibition in neoplastic cells (IC_50_-values: palbociclib 12 nM and 43.9 nM; ribociclib 252.6 nM and 65.5 nM; abemaciclib 13.8 nM and 98.4 nM). In one patient who had received avapritinib, resulting in a partial response (patient #14.2), neoplastic cells also remained sensitive to palbociclib (IC_50_-value: 0.25 nM) and abemaciclib (IC_50_-value: 60.4 nM). These observations suggest that CDK4/CDK6 inhibitors may be suitable drugs for cytoreductive therapy in all patients with AdvSM, regardless of the subtype of disease, the presence of *KIT* D816V, or previous treatment with KIT-targeting drugs. In addition to malignant cell expansion, symptoms caused by mediator release upon MC activation often represent a problem in patients with SM. We were therefore interested to investigate whether CDK4/CKD6 inhibitors are also able to block anti-IgE induced histamine release. In these experiments, high concentrations of palbociclib (10 µM) suppressed anti-IgE-mediated histamine release in basophils (Supplementary Materials, Figure S2). In contrast, ribociclib and abemaciclib failed to block histamine secretion in basophils.

### 3.5. CDK4/CDK6 Inhibitors Induce Apoptosis in CD34+/CD38− Leukemic Stem Cells (LSCs) in AdvSM

**Figure 4:**
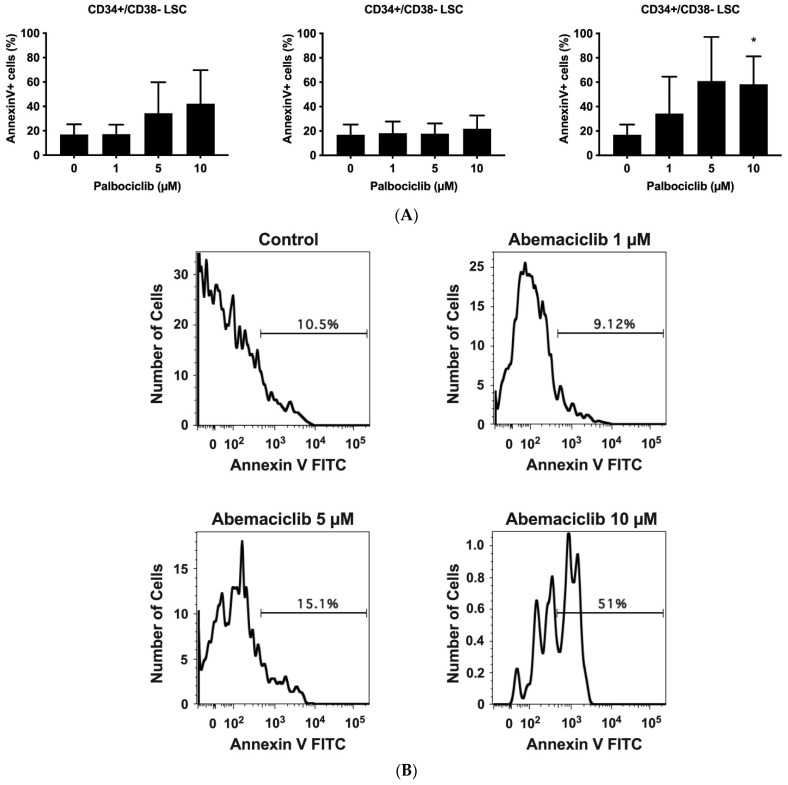
Palbociclib and abemaciclib induce apoptosis in CD34+/CD38− LSC. (**A**): Primary neoplastic BM cells (patient #4.2, #5, #8, and #17 as referred to in Table 1) obtained from patients with AdvSM were exposed to control medium (Co) or palbociclib, ribociclib, or abemaciclib (1 µM, 5 µM, and 10 µM each) for 48 h. Thereafter, CD34+/CD38− LSC were examined for signs of apoptosis by multicolor flow cytometry and staining for Annexin V-FITC and 4′,6-diamidino-2-phenylindole (DAPI). Results represent the mean ± S.D. of three patients (three independent experiments) and are expressed as percentage of AnnexinV+/DAPI− cells (of all CD34+/CD38− cells). (**B**): Histograms of one typical experiment (patient #4.2) are shown. Asterisk (*): *p* < 0.05 compared to control. Abbreviations: LSC, leukemic stem cells; AdvSM, advanced systemic mastocytosis, µM, micromolar.

To achieve long-lasting remissions in AdvSM, it is assumed that eradication of LSCs is necessary. We have recently shown that LSCs in SM exhibit a CD34+/CD38− phenotype [59]. To address whether CDK4/CDK6 inhibitors would counteract the viability of LSCs in the context of AdvSM, we exposed BM samples isolated from patients with ASM or MCL to palbociclib, ribociclib, or abemaciclib and quantified the percentage of apoptotic cells within the CD34+/CD38− compartment. The gating strategy is shown in the Supplementary Materials, Figure S3. In all samples analyzed (patient #4.2, #5, #8, #17 in Table 1), abemaciclib and palbociclib induced apoptosis in LSCs, whereas ribociclib showed only minimal effects on LSCs’ survival in these cells (Figure 4). These observations suggest that abemaciclib, and to a lesser degree palbociclib, exert pro-apoptotic effects on LSCs in AdvSM, which may have clinical implications for patients.

### 3.6. Inhibition of CDK4/CDK6 Sensitizes Neoplastic MCs against Midostaurin

**Figure 5:**
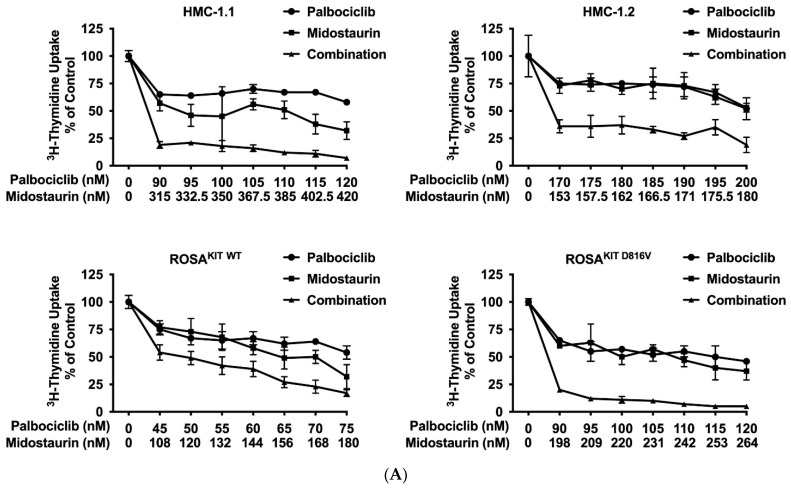

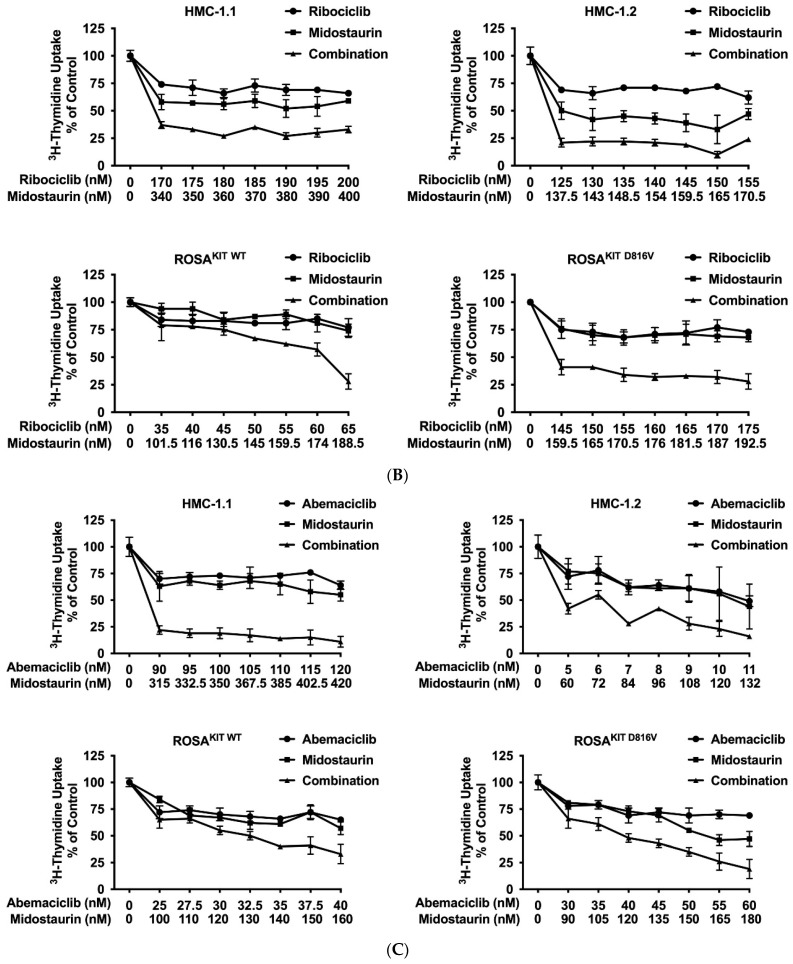

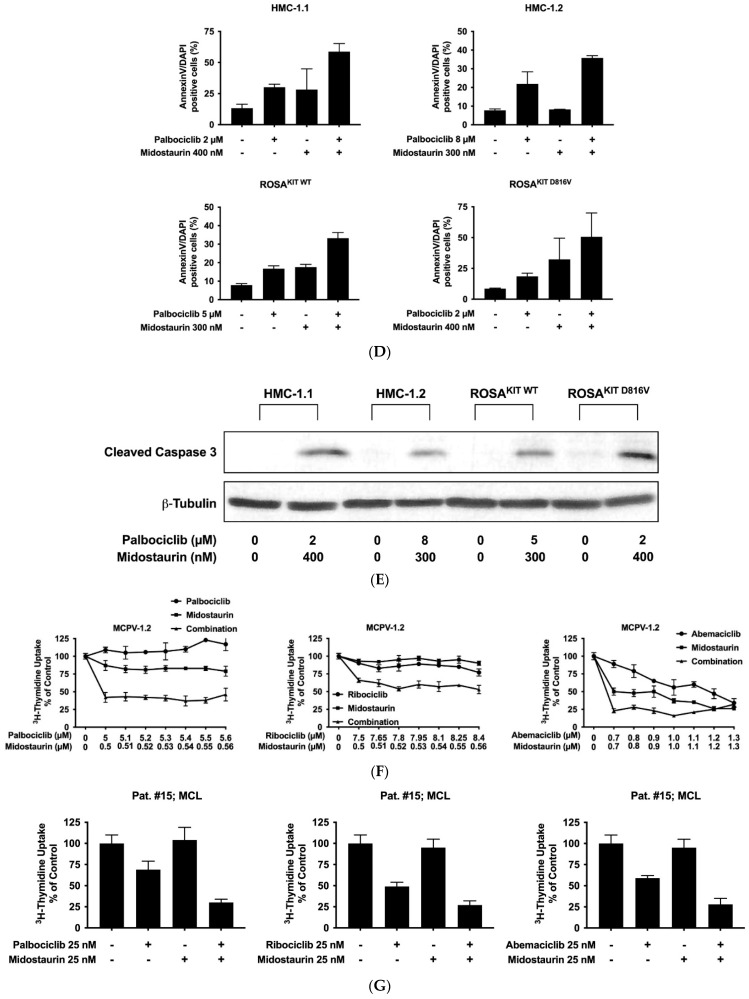
CDK4/CDK6 inhibitors synergize with midostaurin in counteracting proliferation in MC cell lines and primary neoplastic MC. (**A**–**C**) HMC-1 and ROSA cells were incubated with control medium (0 nM) or medium containing various concentrations of palbociclib (**A**), ribociclib (**B**) or abemaciclib (**C**) alone or in combination with midostaurin (as indicated) at 37 °C for 48 h. Thereafter, ^3^H-thymidine uptake was measured in a β-counter. Results are expressed as percentage of control and represent the mean ± S.D from triplicates (**D**) HMC-1 and ROSA cells were incubated with control medium (0 nM) or medium containing various concentrations of palbociclib and/or midostaurin (as indicated) at 37 °C for 72 h. Thereafter, cells were harvested and AnnexinV positive cells were analyzed by flow cytometry. Results show the percentage of apoptotic cells and represent the mean ± S.D. of three independent experiments. (**E**) HMC-1 and ROSA cells were incubated with control medium (Co) or in the presence of palbociclib + midostaurin, as indicated, for 24 h. Thereafter, regulation of cleaved caspase-3 was analyzed by western blotting. (**F**) MCPV-1.2 cells were incubated with control medium (0 µM) or medium containing various concentrations of palbociclib, ribociclib or abemaciclib alone or in combination with midostaurin (as indicated) at 37 °C for 48 h. Thereafter, ^3^H-thymidine uptake was measured. Results are expressed as percentage of control and represent the mean ± S.D from triplicates. (**G**) Primary neoplastic mast cells obtained from a patient with mast cell leukemia (MCL; patient #15 in Table 1) were incubated in control medium or in 25 nM of palbociclib (left panel), ribociclib (middle panel), or abemaciclib (right panel) alone or in combination with midostaurin (25 nM), as indicated, at 37 °C for 48 h. Thereafter, ^3^H-thymidine-uptake was measured. Results are expressed as percentage of control and represent the mean ± S.D from triplicates of one typical experiment. Abbreviations: MCL, mast cell leukemia, nM, nanomolar; µM, micromolar.

Recent data suggest that combinations of targeted drugs may improve the outcome of patients with AdvSM. One reasonable approach consists in combining a KIT D816V-targeting TKI with a drug blocking KIT-independent pathways. Midostaurin is the most widely applied TKI in AdvSM to date. Therefore, we asked whether disruption of the CDK4/CDK6 pathway would increase the anti-neoplastic effects of midostaurin in neoplastic MC. As assessed by ^3^H-thymidine uptake, palbociclib, ribociclib, and abemaciclib were found to cooperate with midostaurin in inhibiting the growth of HMC-1.1, HMC-1.2, ROSA^KIT WT^, and ROSA^KIT D816V^ cells (Figure 5A–C). Additive or synergistic drug effects were confirmed by Calcusyn software and SynergyFinderPlus software (Supplementary Materials, Table S9 and Supplementary Materials, Figure S4A–C). Further, CDK4/CDK6 inhibitors were found to cooperate/synergize with midostaurin in inducing apoptosis in HMC-1 and ROSA cells as assessed by flow cytometry (Figure 5D, Supplementary Materials, Table S10). The induction of apoptosis by the combination ‘palbociclib + midostaurin’ was confirmed by western blotting using an antibody directed against cleaved caspase-3 (Figure 5E). In MCPV-1 cells, which were overall much less sensitive to CDK4/CDK6 inhibitors, all combinations tested produced additive or synergistic anti-proliferative effects (Figure 5F, Supplementary Materials, Table S9, and Supplementary Materials, Figure S4D). Cooperative growth-inhibitory effects between CDK4/CDK6 inhibitors and midostaurin were also confirmed in primary neoplastic cells, as exemplified for one patient with midostaurin-resistant MCL (Figure 5G). These data suggest that all currently available CDK4/CDK6 inhibitors may represent promising combination partners for midostaurin in the treatment of AdvSM.

### 3.7. CDK4/CDK6 Inhibitors Synergize with the New KIT-Targeting Drugs Avapritinib and Nintedanib in Producing Growth Inhibition in Neoplastic MC

**Figure 6:**
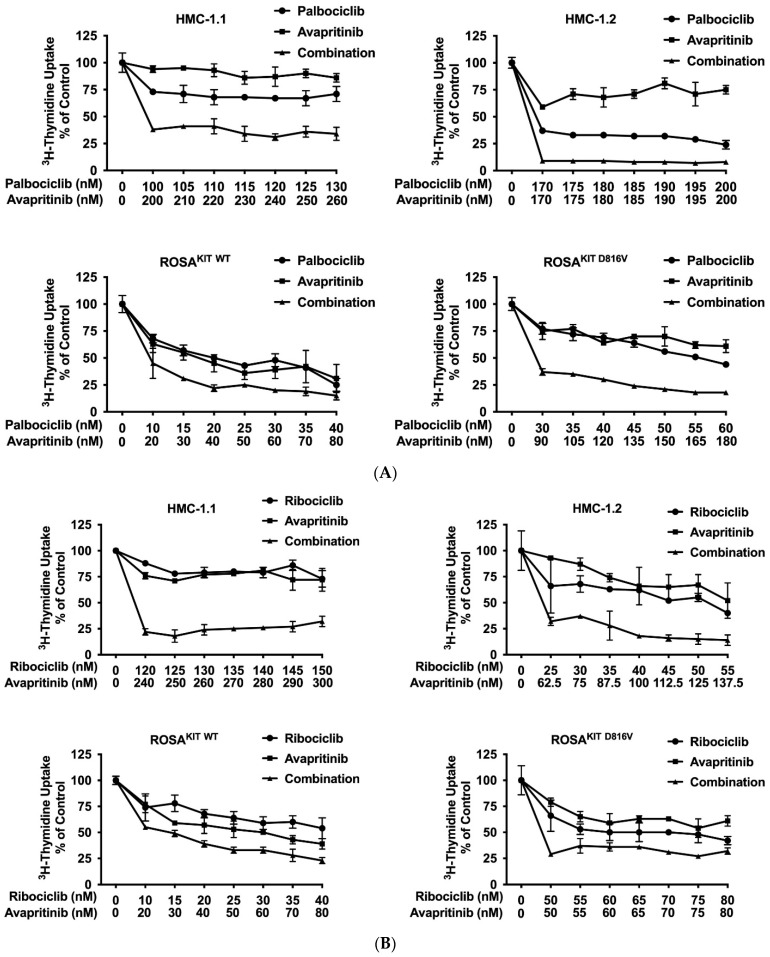

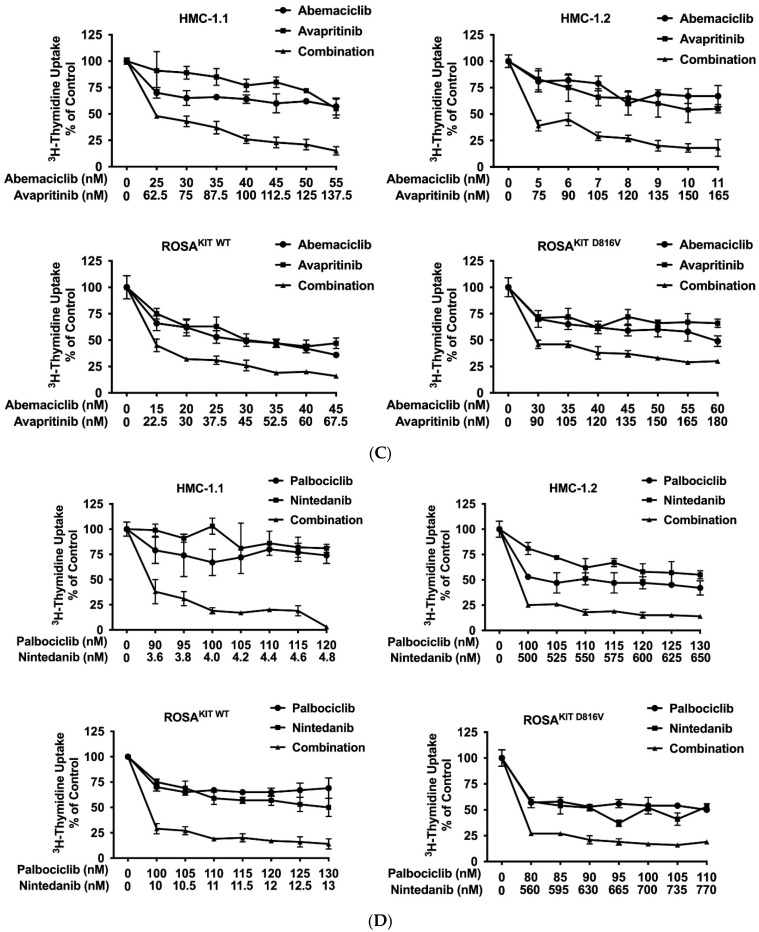

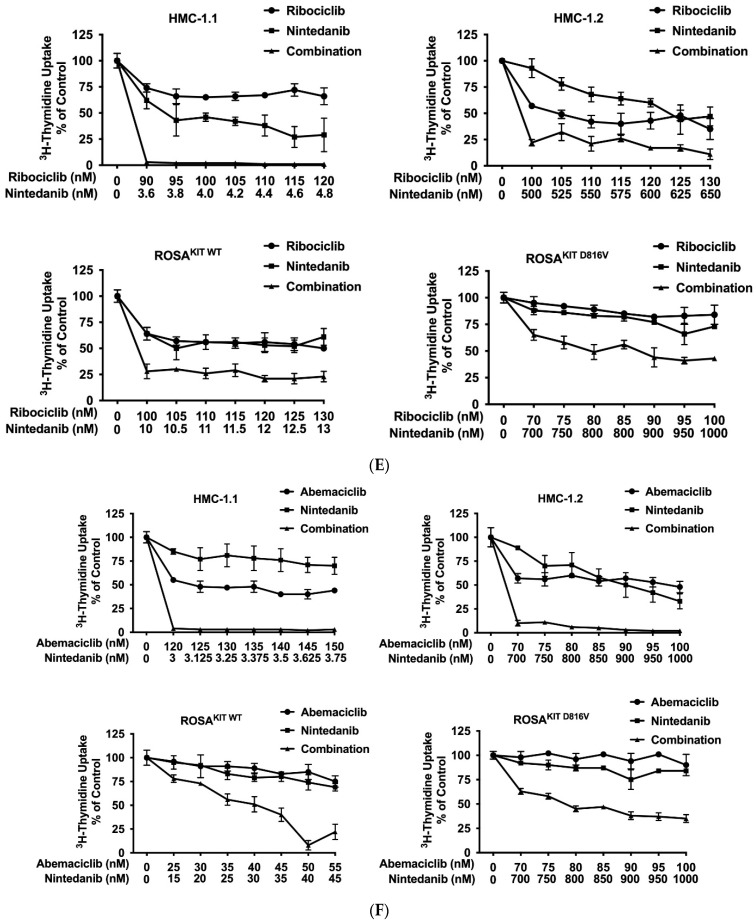

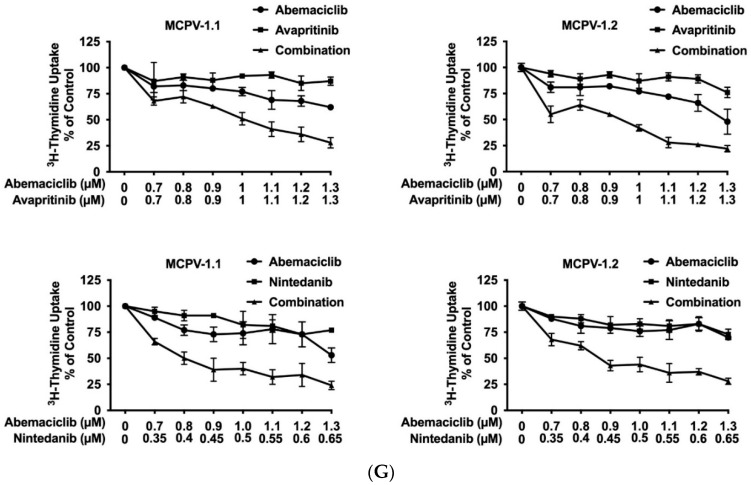
CDK4/CDK6 inhibitors synergize with avapritinib and nintedanib in counteracting proliferation in HMC-1 and ROSA cells. A-C: HMC-1 and ROSA cells were incubated with control medium (0 nM) or medium containing various concentrations of palbociclib (**A**), ribociclib (**B**), or abemaciclib (**C**), alone or in combination with avapritinib, as indicated, at 37 °C for 48 h. (**D**–**F**) HMC-1 and ROSA cells were incubated with control medium (0 nM) or medium containing various concentrations of palbociclib (**D**), ribociclib (**E**), or abemaciclib (**F**), alone or in combination with nintedanib, as indicated, at 37 °C for 48 h. Thereafter, ^3^H-thymidine uptake was measured in a β-counter. (**G**) MCPV-1.1 and MCPV-1.2 cells were incubated with control medium (0 µM) or medium containing various concentrations of abemaciclib alone or in combination with avapritinib (upper panels) or nintedanib (lower panels), as indicated, at 37 °C for 48 h. Thereafter, ^3^H-thymidine uptake was measured in a β-counter. Results (**A**–**G**) are expressed as percentage of control and represent the mean ± S.D from triplicates. Abbreviations: CDK, cyclin-dependent kinase; nM, nanomolar; µM, micromolar.

Current research focuses on the identification and development of KIT D816V-targeting drugs that may be useful in the treatment of AdvSM. Recently, avapritinib, which has been shown to induce major responses in a majority of patients with AdvSM, has gained approval in this indication [20,21,27,28]. Nintedanib has been shown to exert promising results in vitro [67,68]. We examined cooperative antineoplastic effects between CDK4/CDK6 inhibitors and avapritinib/nintedanib. As visible in Figure 6A–F, Supplementary Materials, Table S9, and Supplementary Materials, Figure S5A–F, all three CDK4/CDK6 inhibitors were found to exert synergistic or additive effects with avapritinib and nintedanib in inhibiting the growth of HMC-1.1, HMC-1.2, ROSA^KIT WT^, and ROSA^KIT D816V^ cells. Additive or even synergistic pro-apoptotic effects were confirmed for the combination `abemaciclib + avapritinib´ in HMC-1.2 and ROSA^KIT D816V^ cells (Supplementary Materials, Table S10). In MCPV-1 cells, additive or synergistic effects were also obtained with all combinations tested. As expected, the most potent growth-inhibitory effects were obtained by combining abemaciclib with avapritinib or nintedanib (Figure 6G, Supplementary Materials, Table S9, and Supplementary Materials, Figure S5G).

## 4. Discussion

Midostaurin and avapritinib are KIT-targeting drugs that are used for the treatment of AdvSM and result in a reduction of the MC burden in most patients with AdvSM [14,16,19,20,21,22,23,24,25,26,27,28,29]. However, long-lasting remissions are not observed in all patients, and relapses may occur. This phenomenon may best be explained by the complexity of the disease and by the multiple signaling pathways (other than KIT D816V-dependent pathways) involved in neoplastic MC expansion [22,30,31,33,34,35,36,37]. Therefore, alternative targeted therapies that are effective both as single agents and in combination with midostaurin/avapritinib are warranted. CDK4/CDK6 inhibitors, which are well established for the treatment of breast cancer [39,40,41,42,43,44,45,46,47,48,49], have not previously been analyzed in the context of SM. Here, we show that CDK4 and CDK6 are expressed in neoplastic cells in patients with ISM and AdvSM. Both molecules may play a decisive role in the expansion of neoplastic MC. The three FDA-approved CDK4/CDK6 inhibitors, palbociclib [45,46], ribociclib [47,48], and abemaciclib [39,49,50], were found to block the proliferation of most MCL-like cell lines and primary neoplastic cells in all samples tested. Moreover, abemaciclib, and to a lesser degree palbociclib, induced apoptosis in CD34+/CD38− LSC. Furthermore, CDK4/CDK6 inhibitors were found to sensitize neoplastic MC against the effects of midostaurin, avapritinib, and nintedanib. Together, CDK4/CDK6 inhibitors used as single drugs or in combination with KIT-targeting TKI may be considered for the treatment of AdvSM.

CDK4/CDK6 is a well-established target in applied oncology [39,40,41] and has also been discussed in the context of hematologic malignancies [52,53,54]. However, little attention has been paid so far to the role of this pathway in neoplastic MC. Here, we show that the cell cycle proteins CDK4, CDK6, cyclin D1, cyclin D2, and their downstream molecule RB1, are expressed in BM samples obtained from patients with various forms of SM. Interestingly, the expression of CDKs and cyclins varied among the subvariants of SM. In AdvSM, including SM-AHN, ASM, and MCL, the expression levels of CDK4, CDK6, cyclin D1, cyclin D2, and RB1 in neoplastic cells were slightly higher compared to expression levels in isolated MNC obtained from patients with non-advanced SM (ISM, SSM). It is also worth noting that CDK- and cyclin-expression levels in neoplastic cells varied significantly among patients with AdvSM, without a clear correlation to the subtype of disease. This may be explained by the heterogeneity of patients concerning the burden of MC in the BM, the proliferation of neoplastic cells, or, in the case of SM-AHN, the type of associated disease. Furthermore, independent of the type of SM, CDK4 and CDK6, as well as cyclin D1 and D2, were found to be variably overexpressed in neoplastic cells. Likewise, in some patients with SM-AHN, cyclin D1 was very weakly expressed, whereas CDK4, CDK6, and cyclin D2 were overexpressed compared to ISM. Varying results were also seen in MCL-like cell lines. Interestingly, MCPV-1 cells lacking *KIT* D816V, but expressing *RAS* G12V, Large T and hTert, were found to lack cyclin D2 at the mRNA- and protein level, thereby contrasting all other MCL-like cell lines analyzed. These observations may point to the fact that different pro-oncogenic signaling (involving *KIT* D816V, *RAS* G12V and other mutations) may lead to different expression patterns of CDKs and cyclins. However, which oncogenes may contribute to the overexpression of CDKs and cyclins in neoplastic MCs remains unknown.

CDK4/CDK6 have been described to play an essential role in cell cycle progression, and thus in cell proliferation in normal and neoplastic cells [39,40,41]. However, little is known about the functional role of CDK4/CDK6 in the context of neoplastic MCs. In the current study, we investigated this aspect and found that simultaneous knockdown of both CDK4 and CDK6 leads to growth inhibition in HMC-1.2 cells. These results are in line with previous observations in other cell types and suggest that the CDK4/CDK6 axis is indispensable for cell proliferation, and thus for the expansion of neoplastic MCs.

Within the last decade, the concept of targeting CDK4/CDK6 with suitable drugs has been well established in oncology [39,40,41,42,43]. Indeed, three drugs are currently approved and are used in patients with breast cancer: palbociclib, ribociclib, and abemaciclib [39,44,45,46,47,48,49,50]. In our study, we were able to demonstrate that all three drugs target the CDK4/CDK6 pathway in neoplastic MC. Indeed, pRB1 was downregulated by all drugs in HMC-1 and ROSA cells lacking or expressing *KIT* D816V. Interestingly, total RB1 expression was also decreased in HMC-1 cells after exposure to CDK4/CDK6 inhibitors. In this regard, our data confirm previous observations in other cell types [64,65,66]. So far, no mechanism has been delineated to explain this phenomenon. In our experiments, we did not find evidence of a decreased synthesis of the RB1 protein in HMC-1 cells after exposure to CDK4/CDK6-targeting drugs, at least at the transcriptional level, and increased proteasomal degradation was also eliminated as an underlying mechanism. All in all, the mechanism underlying the suppression of RB1 expression by CDK4/CDK6 inhibitors in HMC-1 cells remains unknown.

In our study, all three CDK4/CDK6 inhibitors were found to induce growth inhibition, cell-cycle arrest, and, to a lesser degree, apoptosis in HMC-1 and ROSA cells expressing or lacking *KIT* D816V at low IC_50_-values. However, in MCPV-1 cells expressing *RAS* G12V, Large T, and hTert, palbociclib and ribociclib failed to produce growth inhibition and a relative resistance against abemaciclib was observed. This is somehow intriguing, as *RAS* G12V has not been associated with resistance against CDK4/CDK6 inhibitors in the context of breast cancer or other malignancies [69]. On the contrary, CDK4/CDK6 inhibitors have even been shown to sensitize *RAS* G12V-mutated cells against chemotherapeutic agents in pancreatic cancer [70]. One explanation for our findings may be that *RAS* G12V mediates resistance against CDK4/CDK6 inhibitors exclusively in the cellular background of neoplastic MC. On the other hand, resistance may also be related to the particular expression pattern of cell-cycle-related kinases in MCPV-1 cells. Indeed, we found that MCPV-1 cells overexpress cyclin D1 but fail to express cyclin D2, contrasting to the other MCL-like cell lines analyzed. Abnormal expression of cyclins D1 and D2 in cancer cells has been discussed as being associated with resistance against CDK4/CDK6 inhibitors [71,72,73]. However, this was largely not confirmed in clinical settings, at least in regard to breast cancer [71].

We next asked whether CDK4/CDK6-targeting drugs would also be able to inhibit the proliferation of primary neoplastic cells and if—by way of analogy to our cell line models—resistance would occur in a subset of samples. In these experiments, all three drugs were found to induce growth inhibition in the nanomolar range in neoplastic cells in all subtypes of SM, including *KIT* D816V^+^ ASM and MCL. The effects of CDK4/CDK6 inhibitors were independent of the presence or absence of an AHN and of the nature of the AHN. These observations suggest that CDK4/CDK6 inhibitors may be suitable drugs for cytoreductive therapy in a majority of patients with AdvSM. Of note, anti-proliferative effects were also confirmed in samples obtained from relapsed cases after midostaurin-treatment and from one patient who underwent treatment with avapritinib. This was predictable to a certain degree, as midostaurin/avapritinib and CDK4/CDK6 inhibitors have different cellular targets and a different mode of action, and no cross-resistances were expected. CDK4/CDK6 inhibitors may, therefore, be interesting drug candidates that may be developed for use as salvage therapy in relapsed drug-resistant AdvSM.

In the recent past, it has been postulated that LSCs, which reside in a CD34+/CD38− cell compartment in AdvSM, may survive conventional or targeted therapy and may, therefore, give rise to disease relapse [60]. To achieve long lasting remissions, it is assumed that eradication of LSCs is necessary [60]. Therefore, current research focusses on identifying drugs or drug combinations that counteract the survival of LSCs in AdvSM. We have found that abemaciclib, and to a lesser degree palbociclib, lead to apoptosis in CD34+/CD38− LSCs obtained from patients with ASM or MCL, suggesting, that these drugs may contribute to eradication of neoplastic MCs and, hence, have a disease-modulating effect. By contrast, the results for ribociclib were rather disappointing in the context of LSCs. Abemaciclib has been shown to be the most effective CDK4/CDK6 inhibitor currently available in different malignant cell types [39,50]. Our study confirms this finding in the context of neoplastic MCs and even LSCs in AdvSM. Whether the superior effect of abemaciclib results from its additional targets [39,50], which may also play a role for the survival of neoplastic MCs and even LSCs in AdvSM, remains unclear so far.

It has been previously observed that patients with AdvSM often fail to achieve long-lasting remissions after treatment with a single drug, such as midostaurin or avapritinib [16,28]. Therefore, drug combinations are considered to represent a more suitable therapeutic approach than treatment with single agents. Here, we showed that CDK4/CDK6 inhibitors synergize with the KIT-inhibitors midostaurin, avapritinib, and nintedanib in producing growth inhibition in MC cell lines and primary neoplastic MCs. The induction of apoptosis in neoplastic MCs may be critical in achieving durable responses in AdvSM. As the pro-apoptotic effects of CDK4/CDK6 inhibitors, when applied alone, were moderate, we asked whether drug combinations (‘CDK4/CDK6-inhibitor + KIT D816V TKI’) would enhance pro-apoptotic effects in neoplastic MC. In our experiments, the percentage of apoptotic cells was significantly increased when CDK4/CDK6 inhibitors were combined with a KIT D816V-targeting TKI, resulting in additive or even synergistic effects, suggesting that such drug combinations may have a more profound antineoplastic efficacy than single drugs. Even in highly resistant MCPV-1 cells, the combination of a CDK4/CDK6 inhibitor with either midostaurin or avapritinib at suboptimal doses resulted in complete growth arrest, suggesting that the drug combination may also be effective in drug-resistant cases of AdvSM.

The implication of CDK4/CDK6 inhibitors—as single drugs or in combination with KIT-targeting TKI—in treatment concepts for AdvSM may have several benefits from a clinical point of view. Palbociclib, ribociclib, and abemaciclib are all approved and therefore available for application in patients. Furthermore, all three drugs are generally well-tolerated when used as single agents. Therefore, these drugs might be used in patients who are resistant or intolerant against midostaurin. The combination “midostaurin + CDK4/CDK6 inhibitor” may result in a more powerful inhibition of neoplastic MC-proliferation than both agents when applied alone. In the case of midostaurin-pretreated ASM/MCL, the combinations “avapritinib + CDK4/CDK6 inhibitor” or “nintedanib + CDK4/CDK6 inhibitor” may be preferable, as these inhibitors show higher affinity to D816V-mutated KIT than midostaurin. Last, two-drug combinations involving a KIT-targeting TKI and a CDK4/CDK6 inhibitor would allow the application of each drug at a lower dose and therefore avoid specific side effects. However, whether these drug combinations would provoke unexpected side effects remains to be carefully determined in upcoming trials.

## 5. Conclusions

Together, our study shows that CDK4 and CDK6 are promising targets in neoplastic MC in AdvSM. The FDA-approved CDK4/CDK6 inhibitors palbociclib, ribociclib, and abemaciclib exert anti-proliferative and pro-apoptotic effects in most MCL cell lines and primary neoplastic MCs and CD34+/CD38− LSC isolated from patients with AdvSM and synergize with KIT-targeting TKIs in producing growth arrest. The value of such drug combinations needs to be evaluated within the frame of upcoming clinical trials.

## Figures and Tables

**Table 1:** Patients’ characteristics (SM patients) and responses of neoplastic cells to CDK4/CDK6 inhibitors.

Patient No.	Age	m/f	Diagnosis, SM Variant	*KIT* D816V	Serum Tryptase ng/mL	BM MC Infiltration %	Palbociclib IC_50_ (nM)	Ribociclib IC_50_ (nM)	Abemaciclib IC_50_ (nM)
#1	61	f	ISM	*+*	25.4	10	12.8	n.t.	n.t.
#2	42	f	ISM	*+*	22.7	2	201.2	148.3	109.2
#3.1	61	m	ISM	*+*	17.5	3	7.2	16.7	4.5
#3.2	62	m	ISM-AML	*+*	26.2	5	40.3	200.2	55.2
#4.1	70	m	ISM	*+*	83.3	20	5.7	n.t.	n.t.
#4.2 *	70	m	ASM	*+*	119	5	94.4	362.1	n.t.
#4.3	70	m	MCL	*+*	94.2	20	12.0	252.6	13.8
#5 *	78	m	ASM-MPN-eo	*+*	45.9	10	67.5	n.t.	n.t.
#6	78	m	ASM-MDS/MPN-u	*+*	101.0	50	4.2	75.4	159.4
#7	75	m	ASM-MDS/MPN-u	*+*	377.0	30	163.4	n.t.	n.t.
#8 *	63	m	ASM-AML	*+*	16.5	10	181.8	n.t.	n.t.
#9	91	m	MCL	*+*	49.6	10	10.4	n.t.	n.t.
#10	86	m	MCL-MDS	*+*	180.0	30	16.8	193.9	23.7
#11	80	f	ISM	*+*	82.9	30	6.5	38.0	19.9
#12	70	m	ASM	*+*	650.0	50	81.6	119.9	135.4
#13	57	m	ISM	*+*	85.4	5	37.0	261.7	16.9
#14.1	70	m	SM-CMML	*+*	374.0	20	2.6	65.8	11.1
#14.2	70	m	SM-CMML	*+*	66.2	2	0.25	408.0	60.4
#15	59	m	MCL	*+*	250.0	60	43.9	65.5	98.4
#16	48	m	SM-CMML	*+*	670.0	60	28.2	134.4	33.3
#17 *	77	m	SM-AML	*+*	30.0	10	n.t.	n.t.	n.t.

Diagnoses were established according to WHO criteria. The percentage of BM MC infiltration was assessed by immunohistochemistry using antibodies against tryptase and KIT. Primary BM mononuclear cells were isolated and incubated with various concentrations of palbociclib, ribociclib, or abemaciclib at 37 °C for 48 h. Then, proliferation was determined by measuring the uptake of ^3^H-thymidine and IC_50_ values were calculated. Abbreviations: WHO, World Health Organization; m: male; f: female; BM, bone marrow; nM, nanomolar; n.t., not tested; IC_50_, half maximal inhibitory concentration; SM, systemic mastocytosis; ISM, indolent SM; ASM, aggressive SM; MCL, mast cell leukemia, AML, acute myeloid leukemia; MDS, myelodysplastic syndrome; MPN, myeloproliferative neoplasm; CMML, chronic myelomonocytic leukemia; u, unspecified; eo, eosinophils. * Patient samples used for stem cell viability assays.

**Table 2:** Effects of CDK4/CDK6 inhibitors on the proliferation of various human mast cell lines.

Cell Line/Cell Type	Palbociclib, IC_50_	Ribociclib, IC_50_	Abemaciclib, IC_50_
HMC-1.1	92.6 ± 10.3 nM	245.6 ± 33.4 nM	174.5 ± 20.9 nM
HMC-1.2	296.1 ± 22.3 nM	703.8 ± 155.0 nM	143.8 ± 14.0 nM
ROSA^KIT WT^	35.1 ± 2.2 nM	262.0 ± 9.5 nM	48.4 ± 3.5 nM
ROSA^KIT D816V^	123.0 ± 13.1 nM	303.3 ± 14.9 nM	134.1 ± 5.1 nM
MCPV-1.1	>10 µM	>10 µM	1.48 ± 0.2 µM
MCPV-1.2	>10 µM	>10 µM	0.9 ± 0.07 µM
MCPV-1.3	>10 µM	>10 µM	2.83 ± 0.43 µM
MCPV-1.4	>10 µM	>10 µM	1.32 ± 0.16 µM

Cell lines were incubated in various concentrations of palbociclib, ribociclib, or abemaciclib at 37 °C for 48 h. Then, proliferation was determined by measuring uptake of ^3^H-thymidine and IC_50_ values were calculated. Values represent the mean ± S.D. from three independent experiments. Abbreviations: IC_50_, half maximal inhibitory concentration; nM: nanomolar; µM, micromolar.

## Data Availability

Data sharing was not applicable.

## Supplementary Materials

The following supporting information can be downloaded at: https://www.mdpi.com/article/10.3390/cancers14133070/s1, Supplemental Methods; Supplemental References; Table S1: Patients’ characteristics (n = 50 patients with SM): samples used for qPCR analysis; Table S2: qPCR primer; Table S3: Antibodies used in western Blot experiments; Table S4: shRNAs applied in knockdown experiments; Table S5: Expression of proteins involved in cell cycle progression in human mast cell lines as analyzed by western blotting; Table S6: Statistical significance of anti-proliferative effects of CDK4/CDK6 inhibitors in the human MC lines HMC-1, ROSA and MCPV-1; Table S7: Statistical significance of pro-apoptotic effects of CDK4/CDK6 inhibitors in the human MC lines HMC-1, ROSA and MCPV-1; Table S8: Statistical significance of anti-proliferative effects of CDK4/CDK6 inhibitors in primary neoplastic MC; Table S9: Cooperative (synergistic or additive) effects between CDK4/CDK6 inhibitors and KIT D816V targeting TKI on proliferation of human MC lines as assessed by ^3^H-thymidine uptake experiments; Table S10: Cooperative (synergistic or additive) effects between CDK4/CDK6 inhibitors and KIT D816V targeting TKI on induction of apoptosis in human MC lines as assessed by flow cytometry; Figure S1A: Detection of CDK4, CDK6, RB1, Cyclin D1 and D2 mRNA in neoplastic cells obtained from patients with non-advanced SM (ISM, SSM) and advanced SM; Figure S1B: Detection of CDK4, CDK6, RB1, Cyclin D1 and D2 mRNA in neoplastic cells in non-advanced SM and advanced SM; Figure S1C: Expression of mRNA transcripts of molecules involved in cell cycle progression in human mast cell lines; Figure S2: Effects of CDK4/CDK6 inhibitors on anti-IgE-induced histamine release in human blood basophils; Figure S3: Gating strategy for identification of mast cells and leukemic stem and progenitor cells; Figure S4A: Synergistic effects of palbociclib and midostaurin applied in combination in MCL-like cell lines as determined by Calcusyn software; Figure S4B: Synergistic effects of ribociclib and midostaurin applied in combination in MCL-like cell lines as determined by Calcusyn software; Figure S4C: Synergistic effects of abemaciclib and midostaurin applied in combination in MCL-like cell lines as determined by Calcusyn software; Figure S4D: Synergistic effects of CDK4/CDK6 inhibitors and midostaurin applied in combination in highly resistant MCPV-1 cells as determined by Calcusyn software; Figure S5A: Synergistic effects of palbociclib and avapritinib applied in combination in MCL-like cell lines as determined by Calcusyn software; Figure S5B: Synergistic effects of ribociclib and avapritinib applied in combination in MCL-like cell lines as determined by Calcusyn software; Figure S5C: Synergistic effects of abemaciclib and avapritinib applied in combination in MCL-like cell lines as determined by Calcusyn software; Figure S5D: Synergistic effects of palbociclib and nintedanib applied in combination in MCL-like cell lines as determined by Calcusyn software; Figure S5E: Synergistic effects of ribociclib and nintedanib applied in combination in MCL-like cell lines as determined by Calcusyn software; Figure S5F: Synergistic effects of abemaciclib and nintedanib applied in combination in MCL-like cell lines as determined by Calcusyn software; Figure S5G: Synergistic effects of abemaciclib and avapritinib applied in combination in highly resistant MCPV-1 cells as determined by Calcusyn software; Additional Information: original western blots and densitometric analysis.
