# Supplementary material for: CDK4/CDK6 Inhibitors Synergize with Midostaurin, Avapritinib, and Nintedanib in Inducing Growth Inhibition in KIT D816V+ Neoplastic Mast Cells
Source: Cancers (Basel). 2022 Jun 23;14(13):3070. doi: 10.3390/cancers14133070 (PMC9264943; doi:10.3390/cancers14133070)
Supplement: Supplementary file 1 [file cancers-14-03070-s001.zip › cancers-1561408-supplementary.pdf]

Supplemental Materials

CDK4/CDK6 inhibitors synergize with midostaurin, avapritinib, and nintedanib in inducing growth-arrest in *KIT* D816V$^+$ neoplastic mast cells

Mathias Schneeweiss-Gleixner[1,2,3], Yüksel Filik[1,2], Gabriele Stefanzl[2], Daniela Berger[2], Irina Sadovnik[1,2], Karin Bauer[1,2], Dubravka Smiljkovic[1,2], Gregor Eisenwort[1,2], Nadine Witzeneder[1], Georg Greiner[1,4,5], Gregor Hoermann[1,6], Ana-Iris Schiefer[7], Juliana Schwaab[8], Mohamad Jawhar[8], Andreas Reiter[8], Wolfgang R. Sperr[1,2], Michel Arock[9], Peter Valent[1,2], Karoline V. Gleixner[1,2]

[1]Ludwig Boltzmann Institute for Hematology and Oncology, Medical University of Vienna, Austria; [2]Department of Internal Medicine I, Division of Hematology and Hemostaseology, Medical University of Vienna, Austria; [3]Department of Medicine III, Division of Gastroenterology and Hepatology, Medical University of Vienna, Austria; [4]Department of Laboratory Medicine, Medical University of Vienna, Austria; [5]Ihr Labor, Medical Diagnostic Laboratories Vienna, Austria; [6]MLL Munich Leukemia Laboratory, Munich, Germany; [7]Department of Pathology, Medical University of Vienna, Austria; [8]Department of Hematology and Oncology, University Medical Center Mannheim and Medical Faculty Mannheim, Heidelberg University, Germany; [9]Department of Hematological Biology, Pitié-Salpêtrière Hospital, Pierre et Marie Curie University (UPMC), Paris, France.

I. Supplemental Methods

Reagents

The dual CDK4/CDK6 inhibitors palbociclib, ribociclib and abemaciclib and the proteasome inhibitor bortezomib were purchased from Selleckchem (Houston, TX, USA). Stock solutions of drugs were prepared by dissolving in dimethyl-sulfoxide (DMSO) (Merck, Darmstadt, Germany). Iscove´s modified Dulbecco´s medium (IMDM) and penicillin/streptomycin were purchased from Lonza (Verviers, Belgium), fetal calf serum (FCS) from Gibco life technologies (Gaithersburg, MD, USA), alpha-thioglycerol and puromycin from Sigma (St. Louis, MO, USA), amphotericin from PAN Biotech (Aidenbach, Germany) and ^{3}H-thymidine from Perkin Elmer (Boston, MA, USA). Propidium Iodide (PI) was obtained from eBioscience (San Diego, CA, USA), AnnexinV/FITC and Annexin V/APC from Biolegend (San Diego, CA, USA). Histamine release buffer (HRB) and histamine radioimmunoassay (RIA) kit were purchased from Immunotech (Marseille, France).

Culture of human cell lines

The human MCL-derived cell line HMC-1 was kindly provided by Joseph H. Butterfield (Mayo Clinic, Rochester, MN, USA) [S1]. Two sub-clones were used, namely HMC-1.1 exhibiting *KIT* V560G, and HMC-1.2 harboring *KIT* V560G and *KIT* D816V. HMC-1 cells were grown in Iscove´s modified Dulbecco´s medium (IMDM) with 10% fetal calf serum (FCS), alpha-thioglycerol and antibiotics at 37°C and 5% CO_2. The human MC lines ROSA$^{KIT\ WT}$ and ROSA$^{KIT\ D816V}$ were generated and cultured essentially as described [S2]. Furthermore, 4 subclones of multi-resistant MCPV-1 cells

[S3] (MCPV-1.1, MCPV-1.2, MCPV-1.3, and MCPV-1.4), lacking *KIT* D816V but expressing *RAS* G12V, Large T and hTert were used in this study. ROSA and MCPV-1 cells were cultured in IMDM containing 10% FCS. In case of ROSA$^{KIT\ WT}$ and MCPV-1 cells, medium was supplemented with stem cell factor (SCF) as described [S1-3].

^{3}H-thymidine incorporation assay

To examine the anti-proliferative effects of CDK4/CDK6 inhibitors primary neoplastic cells and MCL-related cell lines were cultured in 96-well microtiter plates in the absence or presence of various concentrations of palbociclib, ribociclib and abemaciclib for 48 hours. In select experiments, combinations of CDK4/CDK6 inhibitors and KIT-targeting drugs (midostaurin, avapritinib and nintedanib) were applied at a fixed ratio of applied drug concentrations. After treatment, ^{3}H-thymidine was added to each well. Sixteen hours later, cells were harvested on filter membranes (Packard Bioscience, Meriden, CT) in a Filtermate 196 harvester (Packard Bioscience). Filters were air dried and the bound radioactivity was measured in a β-counter (Top-Count NXT, Packard Bioscience). All experiments were performed in triplicates.

Quantitative polymerase chain reaction

To determine mRNA expression levels in BM samples obtained from patients with SM and in the MCL-related cell lines, quantitative polymerase chain reaction (qPCR) was performed. RNA was isolated using the RNeasy Plus Mini Kit (Qiagen, Hilden, Germany). cDNA was synthesized using Moloney murine leukemia virus reverse transcriptase (Invitrogen, Carlsbad, CA, USA), random primers, first strand buffer, dNTPs (100 mM), and RNAsin (all from Promega, Madison, WI, USA) according to

the manufacturer's instructions. PCR was performed as reported [S4] using primers specific for *CDK4*, *CDK6*, *cyclin D1*, *cyclin D2* and *RB1*. A list of PCR primers used in qPCR experiments is provided in Supplemental Table S2. mRNA levels were quantified on a Quant-Studio 3 PCR System (Applied Biosystems, Foster City, CA, USA) using iTAq SYBR Green Supermix with ROX (Bio-Rad, Hercules, CA, USA). mRNA expression was normalized to *ABL1* mRNA and expressed as percentage of *ABL1* mRNA levels. Calculations were based on standard curves established for *CDK4*, *CDK6*, *cyclin D1*, *cyclin D2*, *RB1* and *ABL1* mRNA expression [S4].

shRNA-mediated knockdown of *CDK4* and *CDK6*

HMC-1.2 cells were transfected simultaneously with shRNA constructs directed against *CDK4* (inducible knockdown with doxycycline: basic labeling of shRNA with Venus, and after doxycycline-induced expression, shRNA was detected with dsRed) and *CDK6* (basic labeling of shRNA with Venus, detection of induced shRNA with GFP) or with two non-targeting control shRNAs. For knockdown of *CDK4*, hairpins in a miR-E backbone were cloned into the pRRL-based LT3REVIR vector as described [S5]. For knockdown of *CDK6*, hairpins in a miR-E backbone were cloned into the pRRL-based LT3GEPIR vector as described [S5]. Guide sequences of the hairpins are provided in Supplemental Table S4. Production of recombinant vesicular stomatitis virus G glycoprotein pseudotyped lentiviruses and transduction of target cells were performed as described [S6]. Transduced cells were selected with puromycin (2 µg/ml). Knockdown of *CDK4* and *CDK6* was confirmed by western blot analysis in puromycin selected, doxycycline induced cells. After transfection and incubation with doxycycline, transduced cells were sorted for GFP and dsRed positivity on a BD FACSAria Fusion

(Becton Dickinson, Franklin Lakes, NJ, USA). Un-transduced HMC-1.2 cells were also run through the sorter to provide equal conditions. Thereafter, transduced and un-transduced cells were mixed at a 1:1 ratio and cultured for 10 days. The percentage of GFP+/dsRed+ cells (+/+) was monitored by flow cytometry on days 1, 3, 5 and 10.

Histamine release assay

The histamine release assay was performed on dextran-enriched basophils (healthy donors, n=3) essentially as described [S7-9]. Dextran-enriched basophils were incubated in the presence or absence of various concentrations of CDK4/CDK6 inhibitors (palbociclib, ribociclib, abemaciclib; each 0.01-10 µM) for 30 minutes at 37°C, and thereafter incubated with anti-IgE antibody E124.2.8 (1 µg/mL) in HRB at 37°C for another 30 minutes. After incubation, cells were centrifuged at 4°C, and the cell-free supernatants and total suspensions recovered and analyzed for histamine content by RIA. Histamine release was calculated and expressed as percentage of total histamine. All experiments were performed in triplicates.

II. Supplemental References

S1. Butterfield JH, Weiler D, Dewald G, Gleich GJ. Establishment of an immature mast cell line from a patient with mast cell leukemia. *Leuk Res*. 1988;12(4):345-355.

S2. Saleh R, Wedeh G, Herrmann H, et al. A new human mast cell line expressing a functional IgE receptor converts to factor-independence and tumorigenicity by KIT D816V-transfection. *Blood*. 2014;124(1):111-120.

S3. Hoermann G, Blatt K, Greiner G, et al. CD52 is a molecular target in advanced systemic mastocytosis. *FASEB J*. 2014;28(8):3540-3551.

S4. Stefanzl G, Berger D, Cerny-Reiterer S, et al. The pan-BCL-2-blocker obatoclax (GX15-070) and the PI3-kinase/mTOR-inhibitor BEZ235 produce cooperative growth-inhibitory effects in ALL cells. Oncotarget 2017;28(40):67709–67722.

S5. Fellmann C, Hoffmann T, Sridhar V, et al. An optimized microRNA backbone for effective single-copy RNAi. *Cell Reports*. 2013;5(6):1704-1713.

S6. Hoermann G, Cerny-Reiterer S, Herrmann H, Blatt K, Bilban M, Gisslinger H, et al. Identification of oncostatin M as a JAK2 V617F-dependent amplifier of cytokine production and bone marrow remodeling in myeloproliferative neoplasms. *FASEB J*. 2012;26(2):894-906.

S7. Valent P, Bettelheim P. Cell surface structures on human basophils and mast cells: biochemical and functional characterization. *Adv Immunol*. 1992;52:333-423.

S8. Kneidinger M, Schmidt U, Rix U, et al. The effects of dasatinib on IgE receptor-dependent activation and histamine release in human basophils. *Blood*. 2008;111(6):3097-3107.

S9. Krauth MT, Mirkina I, Herrmann H, Baumgartner C, Kneidinger M, Valent P. Midostaurin (PKC412) inhibits IgE-dependent activation and mediator release in human blood basophils and mast cells. *Clin Exp Allergy*. 2009;39(11):1711-1720.

S10. Eisenwort G, Sadovnik I, Schwaab J, et al. Identification of a leukemia-initiating stem cell in human mast cell leukemia. *Leukemia*. 2019;33(11):2673-2684.

III. Supplemental Tables

Supplemental Table S1

Patients' characteristics (n=50 patients with SM): samples used for qPCR analysis

Patient No.	Gender	Age	Diagnosis	*KIT* D816V	BM Infiltration (%)	Tryptase (µg/L)
S#1	f	29	ISM	+	5	30.5
S#2	m	66	ISM	+	15	65.4
S#3	m	45	ISM	-	5	35.4
S#4	w	56	ISM	-	15	36.6
S#5	w	41	ISM	+	3	17.3
S#6	m	52	ISM	+	10	47.4
S#7	w	32	ISM	+	5	15.5
S#8	w	48	ISM	+	5	36.6
S#9	w	68	ISM	+	5	16.8
S#10	m	38	ISM	+	5	20.1
S#11	w	60	ISM	+	30	144
S#12	w	56	ISM	+	10	43.9
S#13	w	34	ISM	+	5	22
S#14	w	55	ISM	+	30	94.5
S#15	w	78	ISM	+	22	142
S#16	w	63	ISM	+	1	32.7
S#17	m	81	ISM	+	5	30.7
S#18	w	39	ISM	+	30	11.5
S#19	m	30	ISM	+	30	68.2
S#20	w	20	ISM	+	10	41.5
S#21	m	43	ISM	+	30	29.5
S#22	w	22	ISM	+	5	9.2
S#23	w	44	ISM	+	5	14
S#24	w	29	ISM	+	15	45.5
S#25	w	61	ISM	+	25	51.5
S#26	m	24	ISM	+	10	53.7
S#27	m	62	ISM	+	25	26.2
S#28	f	55	SSM	+	20	133
S#29	m	73	ASM	+	25	145
S#30	w	58	ASM	+	20	35
S#31	m	73	ASM	+	60	1926
S#32	m	61	ASM	+	40	401
S#33	m	51	ASM	-	15	36.6
S#34	m	66	SM-AHN	+	15	331
S#35	m	70	SM-AHN	+	5	10.8
S#36	f	78	SM-AHN	+	5	30.4
S#37	m	78	SM-AHN	+	10	118
S#38	m	54	SM-AHN	+	25	284
S#39	m	64	SM-AHN	+	15	247
S#40	m	56	SM-AHN	-	5	16.8
S#41	w	39	SM-AHN	+	15	41.1
S#42	w	64	SM-AHN	-	2	8.9
S#43	m	66	SM-AHN	+	10	186
S#44	m	65	SM-AHN	-	15	37.8

S#45	w	54	MCL	D816H	20	>200
S#46	w	49	MCL	-	70	533
S#47	m	30	MCL	+	20	30
S#48	m	26	MCL	-	30	41.8
S#49	m	58	MCL	+	60	250
S#50	m	73	MCL	+	25	47.2

qPCR was performed with BM samples from 50 patients with SM classified as ISM (n=27), SSM (n=1), ASM (n=5), SM-AHN (n=11) and MCL (n=6). Diagnoses were established according to WHO criteria. The percentage of BM MC infiltration was assessed by immunohistochemistry using antibodies against tryptase and KIT. Abbreviations: No., number; BM, bone marrow; MC, mast cells; WHO, World Health Organization; m, male; f, female; SM, systemic mastocytosis; ISM, indolent SM; SSM, smoldering SM, ASM, aggressive SM; MCL, mast cell leukemia; SM-AHN, SM with an associated hematologic neoplasm.

Supplemental Table S2

qPCR primers

Gene	Sequence
Human CDK4	5´-GTGGACATGTGGAGTGTTGG -3´(fwd)
	5´-CTGGTCGGCTTCAGAGTTTC-3´(rev)
Human CDK6	5´-AAGTTCCAGAGCCTGGAGTG-3´(fwd)
	5´-CGATGCACTACTCGGTGTGA-3´(rev)
Human Cyclin D1	5´-CAAATGTGTGCAGAAGGAGGT-3´(fwd)
	5´-CTCCTCGCACTTCTGTTCCT-3´(rev)
Human Cyclin D2	5´-GTGCAGAAGGACATCCAACC-3´(fwd)
	5´-TCGCACTTCTGTTCCTCACA-3´(rev)
Human ABL1	5´-TGTATGATTTTGTGGCCAGTGGAG-3´(fwd)
	5´-GCCTAAGACCCGGAGCTTTTCA-3´(rev)
Human RB1	5´-AGAGCTTGGTTAACTTGGGAGA-3´(fwd)
	5´-ACAGATTCCCCACAGTTCCTTT-3´(rev)

Abbreviations: qPCR, quantitative polymerase chain reaction analysis; fwd, forward; rev, reversed.

Supplemental Table S3

Antibodies used in western Blot experiments

Protein/Epitope	Clone	Source	Dilution	Company
CDK4	D9G3E	rabbit	1:1000	Cell Signaling
CDK6	DCS83	mouse	1:1000	Cell Signaling
Cyclin D1	92G2	mouse	1:500	Cell Signaling
Cyclin D2	D52F9	mouse	1:750	Cell Signaling
pRB1 (Ser807/811)	polyclonal	rabbit	1:1000	Cell Signaling
RB1	sc-74562	mouse	1:500	Santa Cruz
Cleaved Caspase-3 (Asp175)	polyclonal	rabbit	1:1000	Cell Signaling
Actin	sc-58673	mouse	1:1000	Santa Cruz
β-Tubulin	sc-53140	mouse	1:7500	Santa Cruz

The antibodies were purchased from the following companies: Santa Cruz Biotechnology (Santa Cruz, CA, USA); Cell Signaling Technology (Beverly, MA, USA). Abbreviations: CDK, cyclin dependent kinase; p, phosphorylated; RB1, retinoblastoma protein 1.

Supplemental Table S4

shRNAs applied in knockdown experiments

Gene	Construct Name	Guide sequence
CDK4	CDK4 #3	TAGTGTAGAGAAATGGGAAGGA
CDK6	CDK6 #3	TTTGATAGAGTAAATGTATGGC

Abbreviations: CDK, cyclin dependent kinase

Supplemental Table S5

Expression of proteins involved in cell cycle progression in human mast cell lines as analyzed by western blotting

	HMC-1.1	HMC-1.2	ROSA$^{KIT\ WT}$	ROSA$^{KIT\ D816V}$	MCPV-1.1	MCPV-1.2	MCPV-1.3	MCPV-1.4
**CDK4**	+	+	+	+	+	+	+	+
**CDK6**	+	+	+	+	+	+	+	+
**Cyclin D1**	+	+	+	+	+	+	+	+
**Cyclin D2**	+	+/-	+	+	-	-	-	-
**RB1**	+	+	+	+	+	+	+	+

Mast cell lines were analyzed for expression of cell cycle regulators by western blotting experiments (n=3) using antibodies directed against CDK4, CDK6, cyclin D1, cyclin D2, and RB1. Proteins were either detectable (+) or not detectable (-) in all experiments; in one case, a week protein expression was seen in 2 out of 3 experiments (+/-).

Supplemental Table S6

Statistical significance of anti-proliferative effects of CDK4/CDK6 inhibitors in the human MC lines HMC-1, ROSA and MCPV-1

Cell line	Compound	IC_{50} value	ANOVA	Post-hoc test*
HMC-1.1	Palbociclib	92.6±10.3 nM	p<0.0001	p<0.05 for 0.05-1 µM
HMC-1.1	Ribociclib	245.6±33.4 nM	p<0.0001	p<0.05 for 0.05-1 µM
HMC-1.1	Abemaciclib	174.5±20.9 nM	p<0.0001	p<0.05 for 0.05-1 µM
HMC-1.2	Palbociclib	296.1±22.3 nM	p<0.0001	p<0.05 for 0.1-1 µM
HMC-1.2	Ribociclib	703.8±155 nM	p=0.0014	p<0.05 for 0.5-1 µM
HMC-1.2	Abemaciclib	143.8±14 nM	p<0.0001	p<0.05 for 0.01-1 µM
ROSA $^{KIT\ WT}$	Palbociclib	35.1±2.2 nM	p<0.0001	p<0.05 for 0.01-1 µM
ROSA $^{KIT\ WT}$	Ribociclib	262±9.5 nM	p<0.0001	p<0.05 for 0.05-1 µM
ROSA $^{KIT\ WT}$	Abemaciclib	48.4±3.5 nM	p<0.0001	p<0.05 for 0.01-1 µM
ROSA $^{KIT\ D816V}$	Palbociclib	123±13.1 nM	p<0.0001	p<0.05 for 0.05-1 µM
ROSA $^{KIT\ D816V}$	Ribociclib	303.3±14.9 nM	p<0.0001	p<0.05 for 0.05-1 µM
ROSA $^{KIT\ D816V}$	Abemaciclib	134.1±5.1 nM	p<0.0001	p<0.05 for 0.05-1 µM
MCPV-1.1	Palbociclib	>10 µM	p=0.0953	p<0.05 for 10 µM
MCPV-1.1	Ribociclib	>10 µM	p=0.0021	p<0.05 for 10 µM
MCPV-1.1	Abemaciclib	1.48±0.2 µM	p<0.0001	p<0.05 for 1-10 µM
MCPV-1.2	Palbociclib	>10 µM	p=0.0002	p<0.05 for 10 µM
MCPV-1.2	Ribociclib	>10 µM	p<0.0001	p<0.05 for 0.5-10 µM
MCPV-1.2	Abemaciclib	0.9±0.07 µM	p<0.0001	p<0.05 for 0.05-10 µM
MCPV-1.3	Palbociclib	>10 µM	p=0.0008	p<0.05 for 10 µM
MCPV-1.3	Ribociclib	>10 µM	p<0.0001	p<0.05 for

				10 µM
MCPV-1.3	Abemaciclib	2.83±0.426 µM	p<0.0001	p<0.05 for 10 µM
MCPV-1.4	Palbociclib	>10 µM	p<0.0001	p<0.05 for 10 µM
MCPV-1.4	Ribociclib	>10 µM	p<0.0001	p<0.05 for 10 µM
MCPV-1.4	Abemaciclib	1.32±0.16 µM	p<0.0001	p<0.05 for 5-10 µM

Cell lines were incubated in various concentrations of palbociclib, ribociclib or abemaciclib at 37°C for 48 hours. Then, proliferation was determined by measuring uptake of ^{3}H-thymidine and IC_{50} values were calculated from 3 independent experiments. To determine the significance level in drug-induced inhibition of proliferation, we applied analysis of variance testing (ANOVA). *The post-testing was performed by Dunnett-test; $p<0.05$ is considered statistically significant. Abbreviations: IC_{50}, half maximal inhibitory concentration; nM: nanomolar; µM: micromolar.

Supplemental Table S7

Statistical significance of pro-apoptotic effects of CDK4/CDK6 inhibitors in the human MC lines HMC-1, ROSA and MCPV-1

Cell line	Compound	Range (µM)	ANOVA	Post-hoc test*
HMC-1.1	Palbociclib	1-10	p>0.05	n.a.
HMC-1.1	Ribociclib	1-10	p>0.05	n.a.
HMC-1.1	Abemaciclib	1-10	p>0.05	n.a.
HMC-1.2	Palbociclib	1-10	p>0.05	n.a.
HMC-1.2	Ribociclib	1-10	p>0.05	n.a.
HMC-1.2	Abemaciclib	1-10	p=0.00046	p<0.05 for 5-10 µM
ROSA $^{KIT\ WT}$	Palbociclib	1-10	p>0.05	n.a.
ROSA $^{KIT\ WT}$	Ribociclib	1-10	p>0.05	n.a.
ROSA $^{KIT\ WT}$	Abemaciclib	1-10	p=0.0039	p<0.05 for 5-10 µM
ROSA $^{KIT\ D816V}$	Palbociclib	1-10	p>0.05	n.a.
ROSA $^{KIT\ D816V}$	Ribociclib	1-10	p=0.0301	n.a.
ROSA $^{KIT\ D816V}$	Abemaciclib	1-10	p=0.0002	p<0.05 for 5-10 µM
MCPV-1.1	Palbociclib	1-10	p>0.05	n.a.
MCPV-1.1	Ribociclib	1-10	p>0.05	n.a.
MCPV-1.1	Abemaciclib	1-10	p=0.0073	p<0.05 for 10 µM
MCPV-1.2	Palbociclib	1-10	p>0.05	n.a.
MCPV-1.2	Ribociclib	1-10	p>0.05	n.a.
MCPV-1.2	Abemaciclib	1-10	p=0.0053	p<0.05 for 5-10 µM
MCPV-1.3	Palbociclib	1-10	p>0.05	n.a.
MCPV-1.3	Ribociclib	1-10	p>0.05	n.a.
MCPV-1.3	Abemaciclib	1-10	p>0.05	n.a.
MCPV-1.4	Palbociclib	1-10	p>0.05	n.a.
MCPV-1.4	Ribociclib	1-10	p>0.05	n.a.
MCPV-1.4	Abemaciclib	1-10	p=0.0028	p<0.05 for 5-10 µM

Cell lines were incubated in various concentrations (range: 1–10 µM) of palbociclib, ribociclib or abemaciclib at 37°C for 48 hours. Then, the percentage of apoptotic cells was determined by flow cytometry. To compare the dose effect of different dose levels, we used ANOVA. *Post-testing was performed by Dunnett-test; $p<0.05$ is considered statistically significant. Abbreviations: µM: micromolar; n.a.

Supplemental Table S8

Statistical significance of anti-proliferative effects of CDK4/CDK6 inhibitors in primary neoplastic MC

Patient / Diagnosis	Drug	IC_{50}-value	ANOVA	Post-hoc test**
#1, ISM	Palbociclib	12.8 nM	p<0.0001	p<0.05 for 0.005-2.5 µM
#2, ISM	Palbociclib	201.2 nM	p<0.0001	p<0.05 for 0.125-2.5 µM
#2, ISM	Ribociclib	148.3 nM	p = 0.0138	p<0.05 for 0.25-2.5 µM
#2, ISM	Abemaciclib	109.2 nM	p = 0.02	p<0.05 for 0.005-2.5 µM
#3.1, ISM	Palbociclib	7.2 nM	p<0.0001	p<0.05 for 0.05-2.5 µM
#3.1, ISM	Ribociclib	16.7 nM	p<0.0001	p<0.05 for 0.025-2.5 µM
#3.1, ISM	Abemaciclib	4.5 nM	p<0.0001	p<0.05 for 0.005-2.5 µM
#3.2, ISM-AML	Palbociclib	40.3 nM	p<0.0001	p < 0.05 for 0.025-2.5 µM
#3.2, ISM-AML	Ribociclib	200.2 nM	p = 0.0004	p<0.05 for 0.125-2.5 µM
#3.2, ISM-AML	Abemaciclib	55.2 nM	p<0.0001	p<0.05 for 0.005-2.5 µM
#4.1, ISM	Palbociclib	5.7 nM	p<0.0001	p<0.05 for 0.01-2.5 µM
#4.2, ASM	Palbociclib	94.4 nM	p<0.0001	p< 0.05 for 0.125-2.5 µM
#4.2, ASM	Ribociclib	362.1 nM	p<0.0001	p<0.05 for 0.25-2.5 µM
#5, ASM-MPN-eo	Palbociclib	67.5 nM	p<0.0001	p<0.05 for 0.05-2.5 µM
#6, ASM-MDS/MPN-u	Palbociclib	4.2 nM	p<0.0001	p<0.05 for 0.005-2.5 µM
#6, ASM-MDS/MPN-u	Ribociclib	75.4 nM	p=0.0009	p<0.05 for 0.005-2.5 µM
#6, ASM-MDS/MPN-u	Abemaciclib	159.4 nM	p<0.0001	p<0.05 for 0.005-2.5 µM
#7, ASM-MDS/MPN-u	Palbociclib	163.4nM	p<0.0001	p<0.05 for 0.125-2.5 µM
#8, ASM-AML	Palbociclib	181.8 nM	p=0.0126	p<0.05 for 10 µM
#9, MCL	Palbociclib	10.4 nM	p<0.0001	p<0.05 for 0.005-2.5 µM
#10, MCL-MDS	Palbociclib	16.8 nM	p=0.0073	p<0.05 for 2.5 µM
#10, MCL-MDS	Ribociclib	193.9 nM	p=0.016	p<0.05 for 2.5 µM

#10, MCL-MDS	Abemaciclib	23.7 nM	$p<0.0001$	$p<0.05$ for 0.25-2.5 µM
#11, ISM	Palbociclib	6.5 nM	$p<0.0001$	$p<0.05$ for 0.005-2.5 µM
#11, ISM	Ribociclib	38 nM	$p<0.0001$	$p<0.05$ for 0.005-2.5 µM
#11, ISM	Abemaciclib	19.9 nM	$p<0.0001$	$p<0.05$ for 0.025-2.5 µM
#12, ASM	Palbociclib	81.6 nM	$p<0.0001$	$p<0.05$ for 0.025-2.5 µM
#12, ASM	Ribociclib	119.9 nM	$p<0.0001$	$p<0.05$ for 0.125-2.5 µM
#12, ASM	Abemaciclib	135.4 nM	$p=0.0003$	$p<0.05$ for 0.125-2.5 µM
#13, ISM	Palbociclib	37 nM	$p=0.0004$	$p<0.05$ for 0.125-2.5 µM
#13, ISM	Ribociclib	261.7 nM	$p<0.0001$	$p<0.05$ for 0.5-2.5 µM
#13, ISM	Abemaciclib	16.9 nM	$p<0.0001$	$p<0.05$ for 0.005-2.5 µM
#14.1, SM-CMML	Palbociclib	2.6 nM	$p=0.0003$	$p<0.05$ for 0.005-2.5 µM
#14.1, SM-CMML	Ribociclib	65.8 nM	$p=0.0053$	$p<0.05$ for 0.125-2.5 µM
#14.1, SM-CMML	Abemaciclib	11.1 nM	$p<0.0001$	$p<0.05$ for 0.025-2.5 µM
#14.2, SM-CMML	Palbociclib	0.25 nM	$p<0.0001$	$p<0.05$ for 0.01-2.5 µM
#14.2, SM-CMML	Ribociclib	408 nM	$p<0.0001$	$p<0.05$ for 0.1-2.5 µM
#14.2, SM-CMML	Abemaciclib	60.4 nM	$p<0.0001$	$p < 0.05$ for 0.01-2.5 µM
#15, MCL	Palbociclib	43.9 nM	$p<0.0001$	$p<0.05$ for 0.025-2.5 µM
#15, MCL	Ribociclib	65.5 nM	$p<0.0001$	$p<0.05$ for 0.01-2.5 µM
#15, MCL	Abemaciclib	98.4 nM	$p<0.0001$	$p<0.05$ for 0.025-25 µM
#16, SM-CMML	Palbociclib	28.2 nM	$p<0.0001$	$p<0.05$ for 0.05-2.5 µM
#16, SM-CMML	Ribociclib	134.4 nM	$p<0.0001$	$p < 0.05$ for 2.5 µM
#16, SM-CMML	Abemaciclib	33.3 nM	$p<0.0001$	$p<0.05$ for 0.125-2.5 µM
All samples (n=17*)	Palbociclib	44.9 nM	$p<0.0001$	$p<0.05$ for 0.005-2.5 µM
All samples (n=14)	Ribociclib	158.9 nM	$p<0.0001$	$p<0.05$ for 0.025-2.5 µM
All samples (n=13)	Abemaciclib	57.0 nM	$p<0.0001$	$p<0.05$ for 0.025-2.5 µM

Primary neoplastic cells were incubated in various concentrations of palbociclib, ribociclib or abemaciclib at 37°C for 48 hours. Then, proliferation was determined by measuring uptake of ^{3}H-thymidine and IC_{50} values were calculated from triplicates. Mean values were calculated for all samples tested for each drug (“All samples”; *in case of palbociclib, 3 out of 20 samples tested could not be considered as different drug-concentrations were tested in these samples). To compare the dose effect of different dose levels, we used ANOVA. **Post-testing was performed by Dunnett-test; $p<0.05$ is considered statistically significant. Abbreviations: IC_{50}, half maximal inhibitory concentration; nM: nanomolar; µM: micromolar. Patients´ numbers (#) refer to table 1 in the main manuscript.

Supplemental Table S9

Cooperative (synergistic or additive) effects between CDK4/CDK6 inhibitors and KIT D816V targeting TKI on proliferation of human MC lines as assessed by ^{3}H-thymidine uptake experiments

Cell line	D1 + D2 combination	Range D1 (nM)	Range D2 (nM)	Ratio	CI value	ZIP score
HMC-1.1	palbociclib + midostaurin	90-120	315-200	1:3.5	CI<1 for all conc. tested	15.27
HMC-1.2	palbociclib + midostaurin	170-200	153-180	1:0.9	CI < 1 for `200+180 nM´	14.14
ROSA $^{KIT\ WT}$	palbociclib + midostaurin	45-75	108 -180	1:2.4	CI<1 for all conc. ≥`65+156 nM´	1.96
ROSA $^{KIT\ D816V}$	palbociclib + midostaurin	90-120	198-264	1:2.2	CI<1 for all conc. tested	17.52
HMC-1.1	ribociclib + midostaurin	170-200	340-400	1:2	CI<1 for all conc. tested	6.52
HMC-1.2	ribociclib + midostaurin	125-155	137.5-170.5	1:1.1	CI<1 for all conc. tested	6.98
ROSA $^{KIT\ WT}$	ribociclib + midostaurin	35-65	101.5-188.5	1:2.9	CI<1 for all conc. ≥`45+130.5 nM´	3.08
ROSA $^{KIT\ D816V}$	ribociclib + midostaurin	145-175	159.5-192.5	1:1.1	CI<1 for all conc. tested	19.28
HMC-1.1	abemaciclib + midostaurin	90-120	315-420	1:3.5	CI<1 for all conc. tested	27.12
HMC-1.2	abemaciclib + midostaurin	5-11	60-132	1:12	CI<1 for all conc. ≥`7+84 nM´	2.11
ROSA $^{KIT\ WT}$	abemaciclib + midostaurin	25-40	100-160	1:4	CI<1 for all conc. ≥`35+140 nM´	-3.62
ROSA $^{KIT\ D816V}$	abemaciclib + midostaurin	30-60	90-180	1:3	CI<1 for all conc. ≥`40+120 nM´	15.67
MCPV-1.1	palbociclib + midostaurin	5000-5600	500-560	1:0.1	CI<1 for all conc. tested	2.64
MCPV-1.2	palbociclib + midostaurin	5000-5600	500-560	1:0.1	CI<1 for all conc. tested	49.13
MCPV-1.1	ribociclib + midostaurin	7500-8400	500-560	15:1	CI<1 for all conc. tested	18.35
MCPV-1.2	ribociclib + midostaurin	7500-8400	500-560	15:1	CI<1 for `780+520 nM´	20.5
MCPV-1.1	abemaciclib + midostaurin	700-1300	700-1300	1:1	CI<1 for `700+700 nM´	3.18
MCPV-1.2	abemaciclib + midostaurin	700-1300	700-1300	1:1	CI<1 for `700+700 nM´	-0.76
HMC-1.1	palbociclib + avapritinib	100-130	200-260	1:2	CI<1 for all conc. tested	24.07
HMC-1.2	palbociclib + avapritinib	170-200	170-200	1:1	CI<1 for all conc. tested	14.76

ROSA $^{KIT\ WT}$	palbociclib + avapritinib	10-40	20-80	1:2	CI<1 for all conc. tested	-4.06
ROSA $^{KIT\ D816V}$	palbociclib + avapritinib	30-60	90-180	1:3	CI<1 for all conc. tested	14.60
HMC-1.1	ribociclib + avapritinib	120-150	240-300	1:2	CI<1 for all conc. tested	33.42
HMC-1.2	ribociclib + avapritinib	25-55	62.5-137.5	1:2.5	CI<1 for all conc. tested	14.87
ROSA $^{KIT\ WT}$	ribociclib + avapritinib	10-40	20-80	1:2	CI<1 for all conc. tested	-4.99
ROSA $^{KIT\ D816V}$	ribociclib + avapritinib	50-80	50-80	1:1	CI<1 for all conc. ≥`65+65 nM´	-2.41
HMC-1.1	abemaciclib + avapritinib	25-55	62.5-137.5	1:2.5	CI<1 for all conc. tested	14.24
HMC-1.2	abemaciclib + avapritinib	5-11	75-165	1:15	CI<1 for all conc. tested	14.52
ROSA $^{KIT\ WT}$	abemaciclib + avapritinib	15-45	22.5-67.5	1:1.5	CI<1 for all conc. tested	-0.59
ROSA $^{KIT\ D816V}$	abemaciclib + avapritinib	30-60	90-180	1:3	CI<1 for all conc. tested	1.83
HMC-1.1	palbociclib + nintedanib	90-120	3.6-4.8	1:0.04	CI<1 for all conc. tested	46.72
HMC-1.2	palbociclib + nintedanib	100-130	500-650	1:5	CI<1 for all conc. ≥`110+550 nM´	11.6
ROSA $^{KIT\ WT}$	palbociclib + nintedanib	100-130	10-13	1:0.1	CI<1 for all conc. tested	22.21
ROSA $^{KIT\ D816V}$	palbociclib + nintedanib	80-110	560-770	1:7	CI<1 for all conc. tested	6.47
HMC-1.1	ribociclib + nintedanib	90-120	3.6-4.8	1:0.04	CI<1 for all conc. tested	27.86
HMC-1.2	ribociclib + nintedanib	100-130	500-650	1:5	CI>1 for all conc. tested	7.03
ROSA $^{KIT\ WT}$	ribociclib + nintedanib	100-130	10-13	1:0.1	CI<1 for all conc. tested	7.54
ROSA $^{KIT\ D816V}$	ribociclib + nintedanib	70-100	700-1000	1:10	CI>1 for all conc. tested	17.68
HMC-1.1	abemaciclib + nintedanib	120-150	3-3.75	1:0.025	CI<1 for all conc. tested	32.03
HMC-1.2	abemaciclib + nintedanib	70-100	700-1000	1:10	CI<1 for all conc. tested	26.51
ROSA $^{KIT\ WT}$	abemaciclib + nintedanib	25-55	15-45	1:0.6	CI<1 for all conc. tested	21.57
ROSA $^{KIT\ D816V}$	abemaciclib + nintedanib	70-100	700-1000	1:10	CI<1 for all conc. ≤`100+1000 nM´	38.33
MCPV-1.1	abemaciclib + avapritinib	700-1300	700-1300	1:1	CI<1 for all conc. tested	11.99
MCPV-1.2	abemaciclib + avapritinib	700-1300	700-1300	1:1	CI<1 for all conc. tested	18.28
MCPV-1.3	abemaciclib + avapritinib	700-1300	700-1300	1:1	CI<1 for all conc. tested	11.94

MCPV-1.4	abemaciclib + avapritinib	700-1300	700-1300	1:1	CI<1 for all conc. ≥ `900+900 nM´	16.20
MCPV-1.1	abemaciclib + nintedanib	700-1300	350-650	0:0.5	CI<1 for all conc. ≥`800+400 nM´	21.44
MCPV-1.2	abemaciclib + nintedanib	700-1300	350-650	0:0.5	CI<1 for all conc. tested	18.99
MCPV-1.3	abemaciclib + nintedanib	700-1300	350-650	0:0.5	CI<1 for all conc. ≥`900+450 nM´	9.28
MCPV-1.4	abemaciclib + nintedanib	700-1300	350-650	0:0.5	CI<1 for all conc. tested	22.86

For evaluation of potential cooperative (synergistic or additive) drug-effects, cell lines were incubated in various two-drug-combinations (as described in the main document) each involving a CDK4/CDK6 inhibitor (palbociclib, ribociclib or abemaciclib) and a KIT D816V-TKI (midostaurin. avapritinib or nintedanib) at a fixed ratio. Proliferation was determined by measuring uptake of ^{3}H-thymidine. Drug interaction-types were determined by 2 different methods. Combination index (CI) values were determined using Calcusyn software. A CI of <1 indicates a synergistic effect, whereas as CI of >1 indicates antagonism. Furthermore, the type of drug combination effects was analyzed by calculating synergy scores (zero interaction potency, ZIP) by SynergyfinderPlus software. A synergy score >10 stands for synergism, a synergy score between -10 and 10 for an additive effect and a synergy score <-10 stands for antagonism. Abbreviations: D: drug; nM: nanomolar; conc.: concentrations; CI: combination index; ZIP: zero interaction potency.

Supplemental Table S10

Cooperative (synergistic or additive) effects between CDK4/CDK6 inhibitors and KIT D816V targeting TKI on induction of apoptosis in human MC lines as assessed by flow cytometry

Cell line	D1	D2	Range D1 (µM)	Range D2 (µM)	ZIP score
HMC-1.1	palbociclib	midostaurin	0.5-2.0	0.3-0.5	15.68
HMC-1.2	palbociclib	midostaurin	4.0-8.0	0.3-0.5	11.63
ROSA $^{KIT\ WT}$	palbociclib	midostaurin	1.0-5.0	0.3-0.5	-5.21
ROSA $^{KIT\ D816V}$	palbociclib	midostaurin	0.5-2.0	0.1-0.4	4.93
HMC-1.2	abemaciclib	midostaurin	2.0-4.0	0.3-0.5	25.41
HMC-1.2	abemaciclib	avapritinib	2.0-4.0	0.3-0.5	14.54
ROSA $^{KIT\ D816V}$	abemaciclib	midostaurin	2.0-3.0	0.3-0.5	-0.46
ROSA $^{KIT\ D816V}$	abemaciclib	avapritinib	2.0-3.0	0.5-0.7	4.07

For evaluation of potential cooperative (synergistic or additive) drug-effects on induction of apoptosis, cell lines were incubated in various two-drug-combinations involving each a CDK4/CDK6 inhibitor (palbociclib or abemaciclib) and a KIT D816V-TKI (midostaurin or avapritinib) at 37°C for 72 hours. Then, the percentage of apoptotic cells was determined by flow cytometry. Furthermore, the type of drug combination effects was analyzed by calculating synergy scores (zero interaction potency, ZIP) by SynergyfinderPlus software. A ZIP score >10 stands for synergism, a ZIP score between -10 and 10 for an additive effect and a ZIP score <-10 stands for antagonism. Abbreviations: D: drug; µM: micromolar.

IV. Supplemental Figures

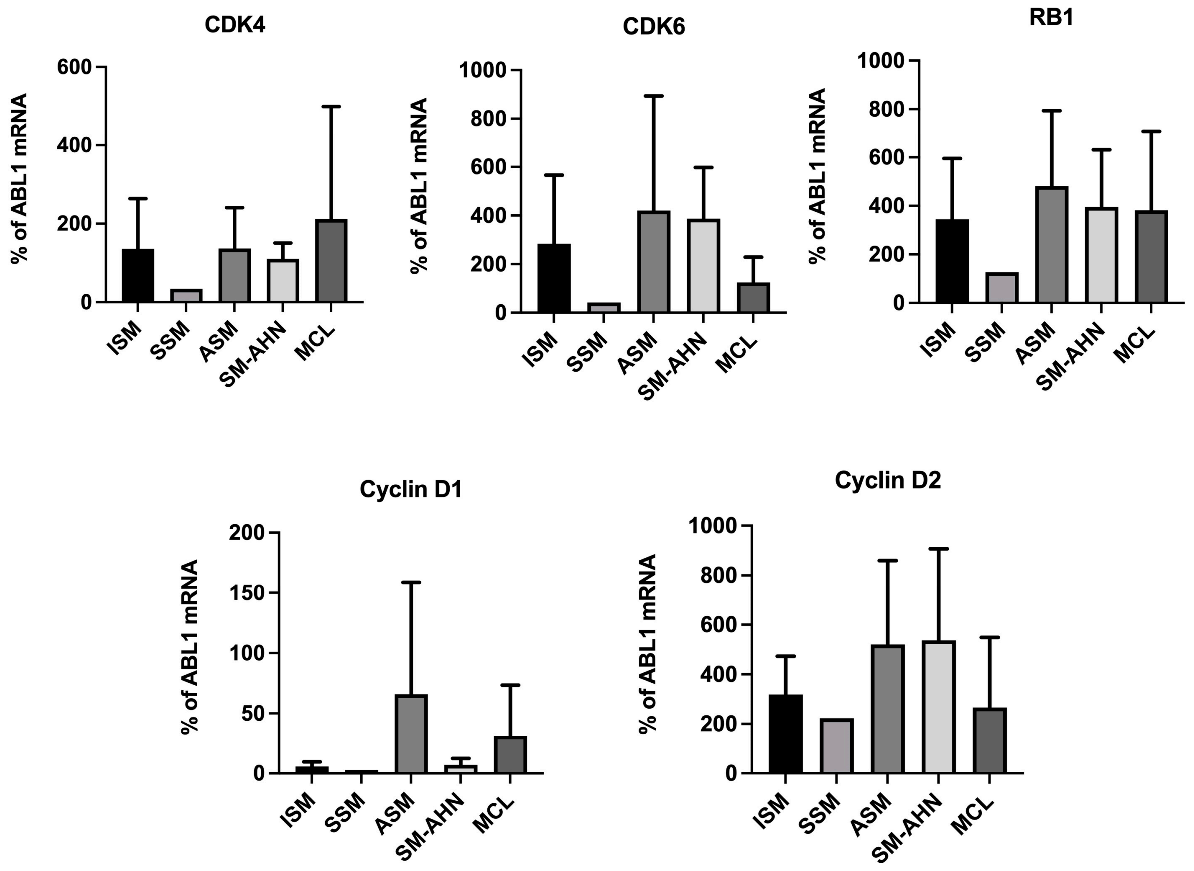

Schneeweiss-Gleixner et al., Supplemental Figure S1A

Detection of CDK4, CDK6, RB1, Cyclin D1 and D2 mRNA in neoplastic cells obtained from patients with non-advanced SM (ISM, SSM) and advanced SM. Primary neoplastic cells (mononuclear cells enriched with Ficoll) from the bone marrow of 50 patients with SM (ISM: n=27; SSM: n=1; ASM: n=5; SM-AHN: n=11; MCL: n=6) patients were subjected to RNA isolation and examined for expression of CDK4, CDK6, RB1, Cyclin D1 and Cyclin D2 mRNA by qPCR. Transcript levels were calculated as percent of ABL1 mRNA. Abbreviations: SM, systemic mastocytosis; ISM, indolent SM; SSM, smoldering SM; ASM, aggressive SM; SM-AHN, SM with an associated hematologic neoplasm; MCL, mast cell leukemia; CDK, cyclin-dependent kinase; RB1, retinoblastoma protein 1.

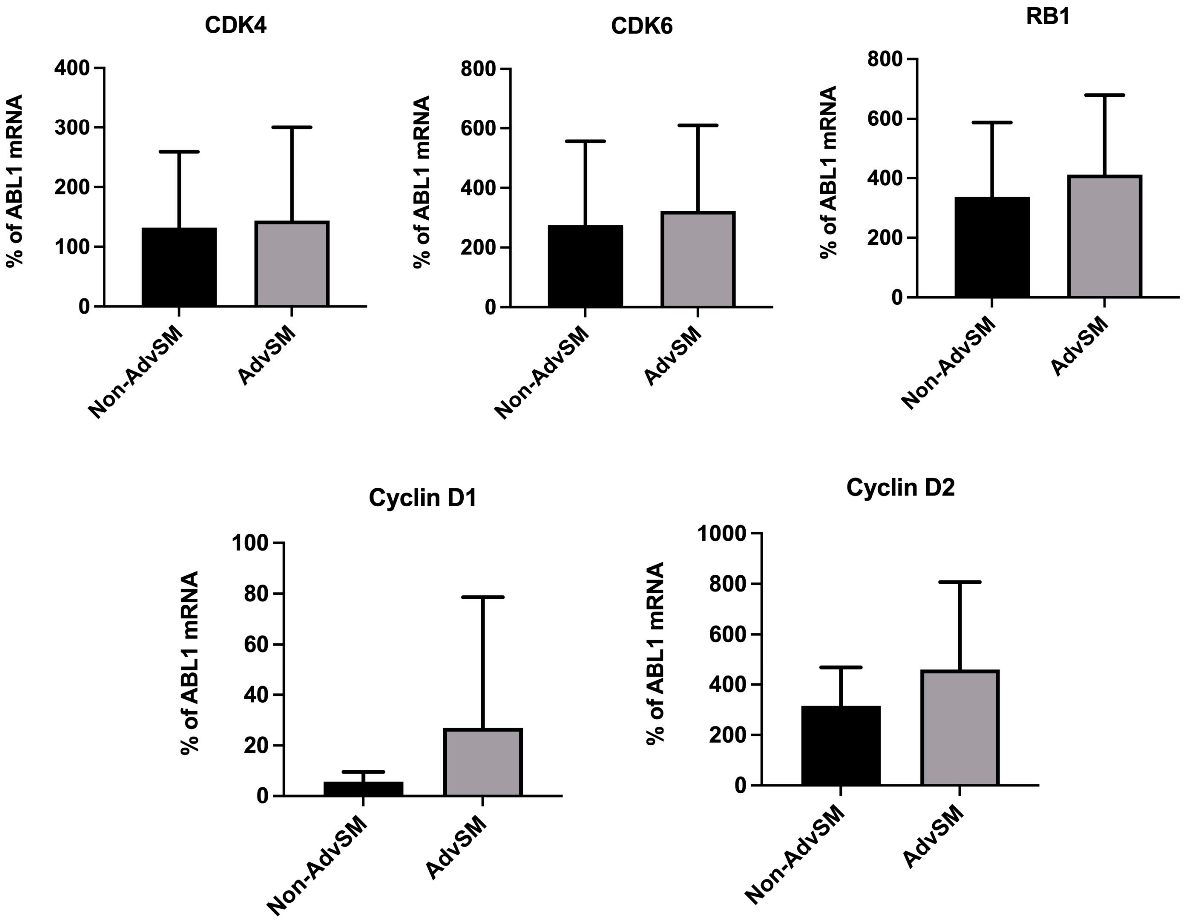

Schneeweiss-Gleixner et al., Supplemental Figure S1B

Detection of CDK4, CDK6, RB1, Cyclin D1 and D2 mRNA in neoplastic cells in non-advanced SM and advanced SM.

Primary neoplastic cells from the bone marrow of 50 patients with SM (ISM: n=27; SSM: n=1; ASM: n=5; SM-AHND: n=11; MCL: n=6) were subjected to RNA isolation and examined for expression of Cyclin D1, Cyclin D2, CDK4, and CDK6 mRNA levels by qPCR. Transcript levels were calculated as percent of ABL1 mRNA. Patient were categorized as non-advanced SM (Non-AdvSM: ISM and SSM) and advanced SM (AdvSM: ASM, SM-AHN, MCL). Abbreviaitons: SM, systemic mastocytosis; ISM, indolent SM; SSM, smoldering SM; ASM, aggressive SM; SM-AHN, SM with an associated hematologic neoplasm; MCL, mast cell leukemia; CDK, cyclin-dependent kinase; RB1, retinoblastoma protein 1.

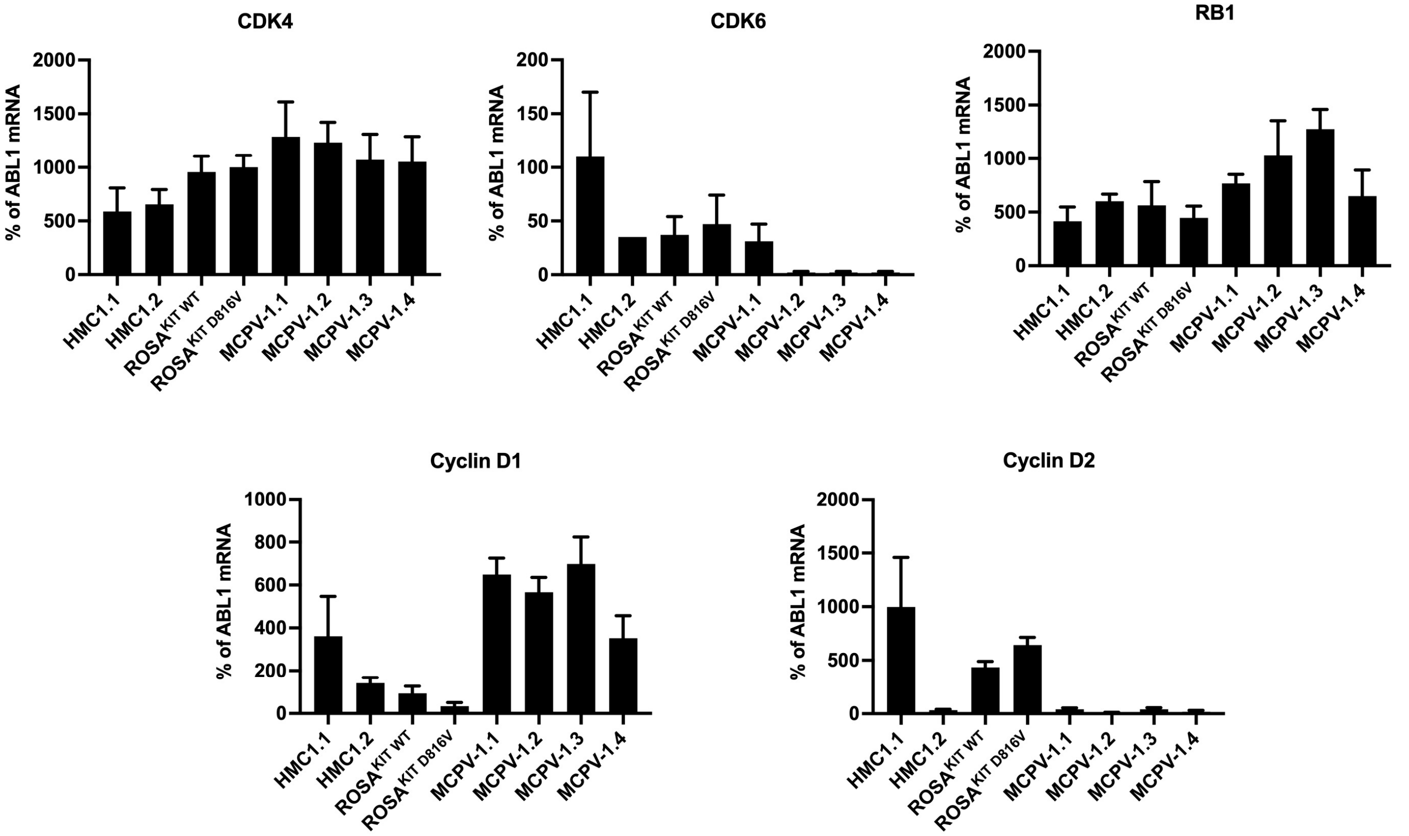

Schneeweiss-Gleixner et al., Supplemental Figure S1C

Expression of mRNA transcripts of molecules involved in cell cycle progression in human mast cell lines.

Mast cell lines (HMC-1, ROSA and MCPV-1) (B) were subjected to RNA isolation and examined for expression of Cyclin D1, Cyclin D2, CDK4 and CDK6 mRNA by qPCR. Transcript levels were calculated and expressed as percent of ABL1 mRNA. Abbreviations: CDK, cyclin-dependent kinase; RB1, retinoblastoma protein 1.

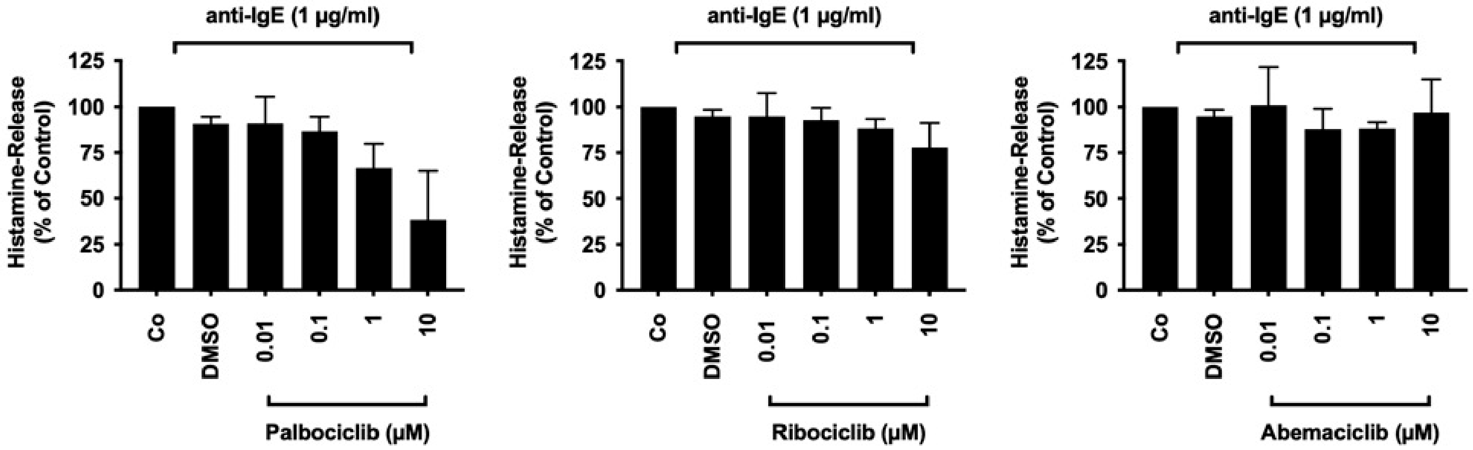

Schneeweiss-Gleixner et al., Supplemental Figure S2

Effects of CDK4/CDK6 inhibitors on anti-IgE-induced histamine release in human blood basophils.

Primary blood basophils obtained from healthy donors were incubated in control medium or in various concentrations of palbociclib, ribociclib, or abemaciclib as indicated, at 37°C for 30 minutes. Thereafter, cells were incubated in control buffer or in buffer containing anti-IgE antibody E-124.2.8 (1 µg/ml) at 37°C for 30 minutes. After incubation, cells were centrifuged at 4°C, and cell-free supernatants and cell suspensions recovered and examined for histamine content by RIA. Histamine release was calculated as percent of total (cellular+extracellular) histamine and is expressed as percent of control.

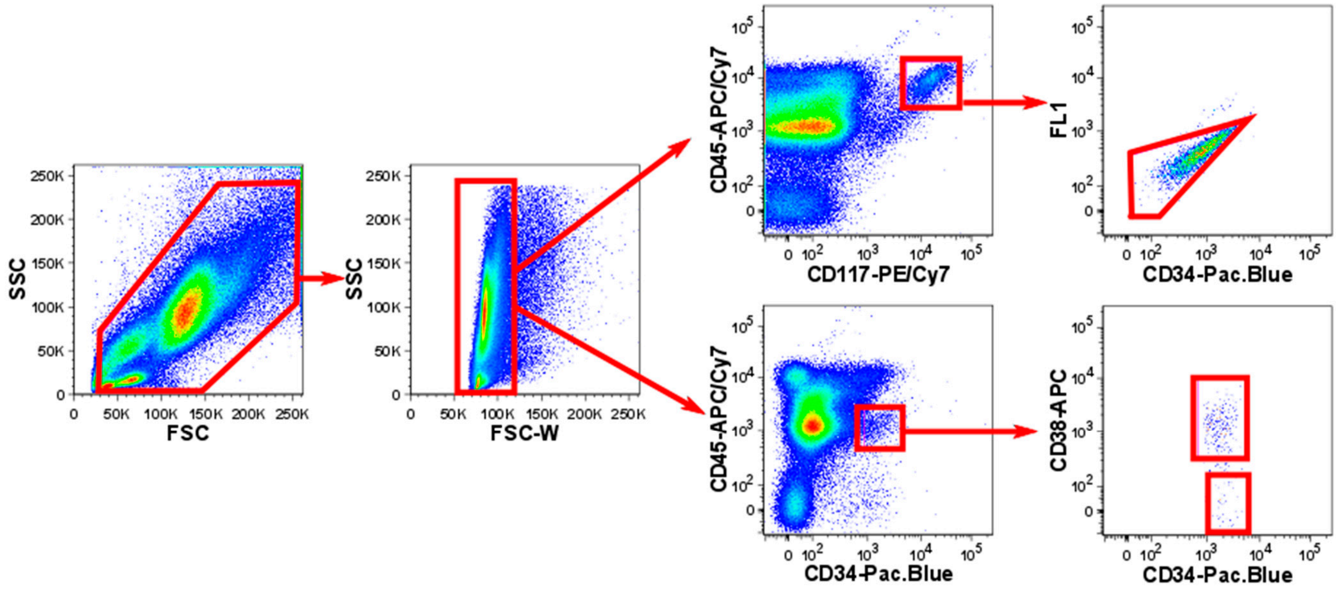

Schneeweiss-Gleixner et al., Supplemental Figure S3

Gating strategy for identification of mast cells and leukemic stem and progenitor cells.

Heparinized bone marrow cells (BM) were obtained from 4 patients with advanced SM (ASM, n=1; ASM-AHN, n=3). The figure shows an example of gating of BM cells in a patient with ASM where the bulk of leukemic cells was analyzed for visualization of KIT+ mast cells and of CD34+ stem- and progenitor cells. The sequential gating strategy is indicated by red arrows. First, non-viable cells and doublets were excluded to define pure populations of viable cells (first and second panel from left). Mast cells were identified by their expression of CD45 and KIT; in a next step, auto-fluorescent cells and CD34+ (stem and progenitor) cells were excluded (upper right panel). Stem- and progenitor cells were gated as CD34+ and CD45 dim-positive cells (lower panels). Thereafter, cells were selected according to their expression of CD38 (progenitor cells) or lack of CD38 (stem cells) (lower right panel). Abbreviations: APC, allophycocyanin; Cy7, Cyanine-7; PE, phycoerythrin; Pac. Blue, Pacific Blue; FSC, forward scatter; FSC-W, FSC signal-width; SSC, side scatter. Figure adapted from Eisenwort et. al, Leukemia 2019 (supplemental reference S10).

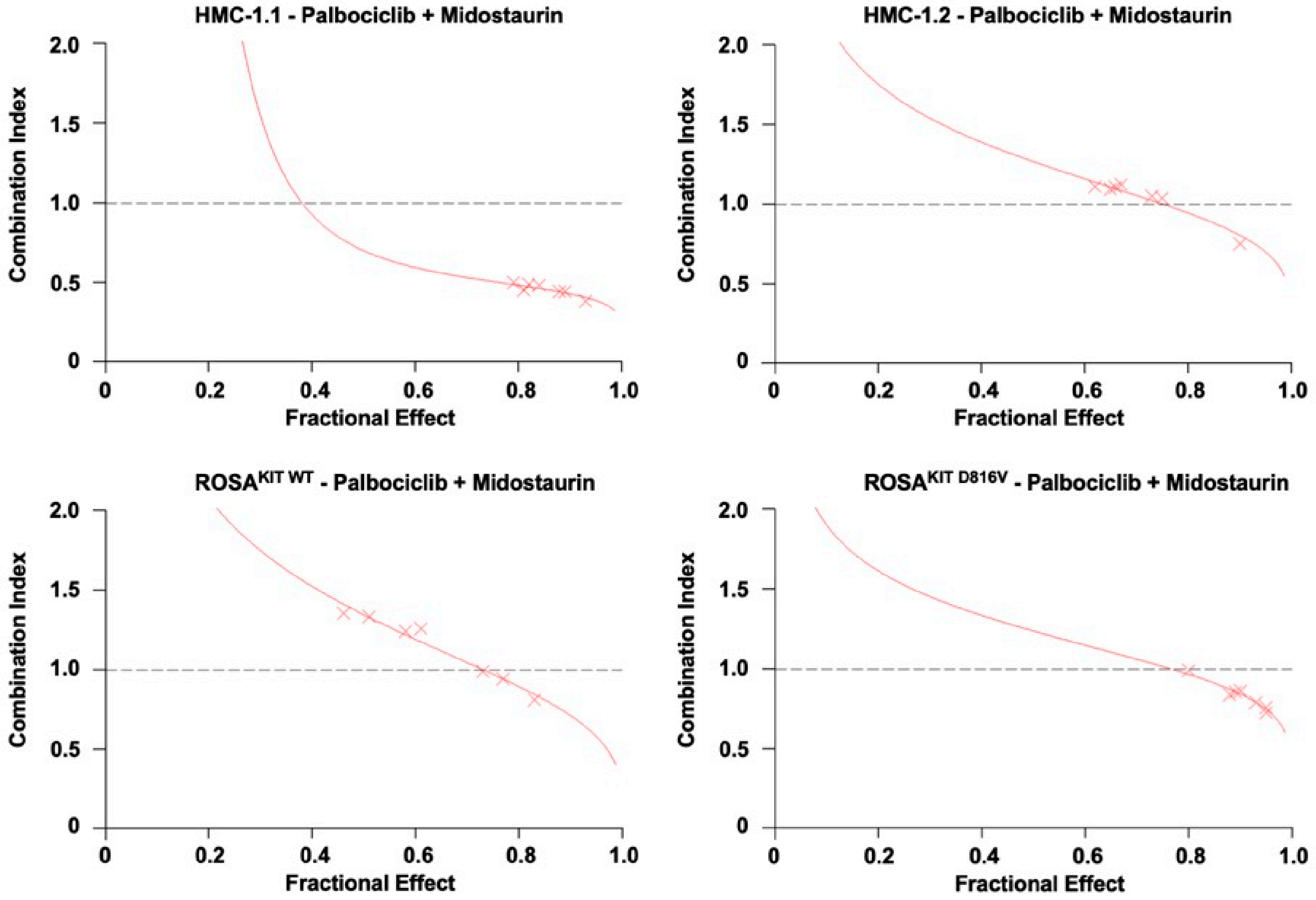

Schneeweiss-Gleixner et al., Supplemental Figure S4A

Synergistic effects of palbociclib and midostaurin applied in combination in MCL-like cell lines as determined by Calcusyn software

HMC-1.1, HMC-1.2, ROSA$^{KIT\ WT}$ and ROSA$^{KIT\ D816V}$ were kept in control medium or in various concentrations of palbociclib, midostaurin or a combination of both drugs at a fixed ratio at 37°C for 48 hours. Thereafter, cell proliferation was determined by measuring ^{3}H-thymidine uptake as shown in Figure 5A in the main document. The nature of drug interaction (additive versus synergistic) was determined for each experiment by calculating combination index (CI) values using Calcusyn software. The resulting CI values are shown. A CI value of 1 indicates an additive effect, whereas CI values below 1 indicate synergistic drug effects.

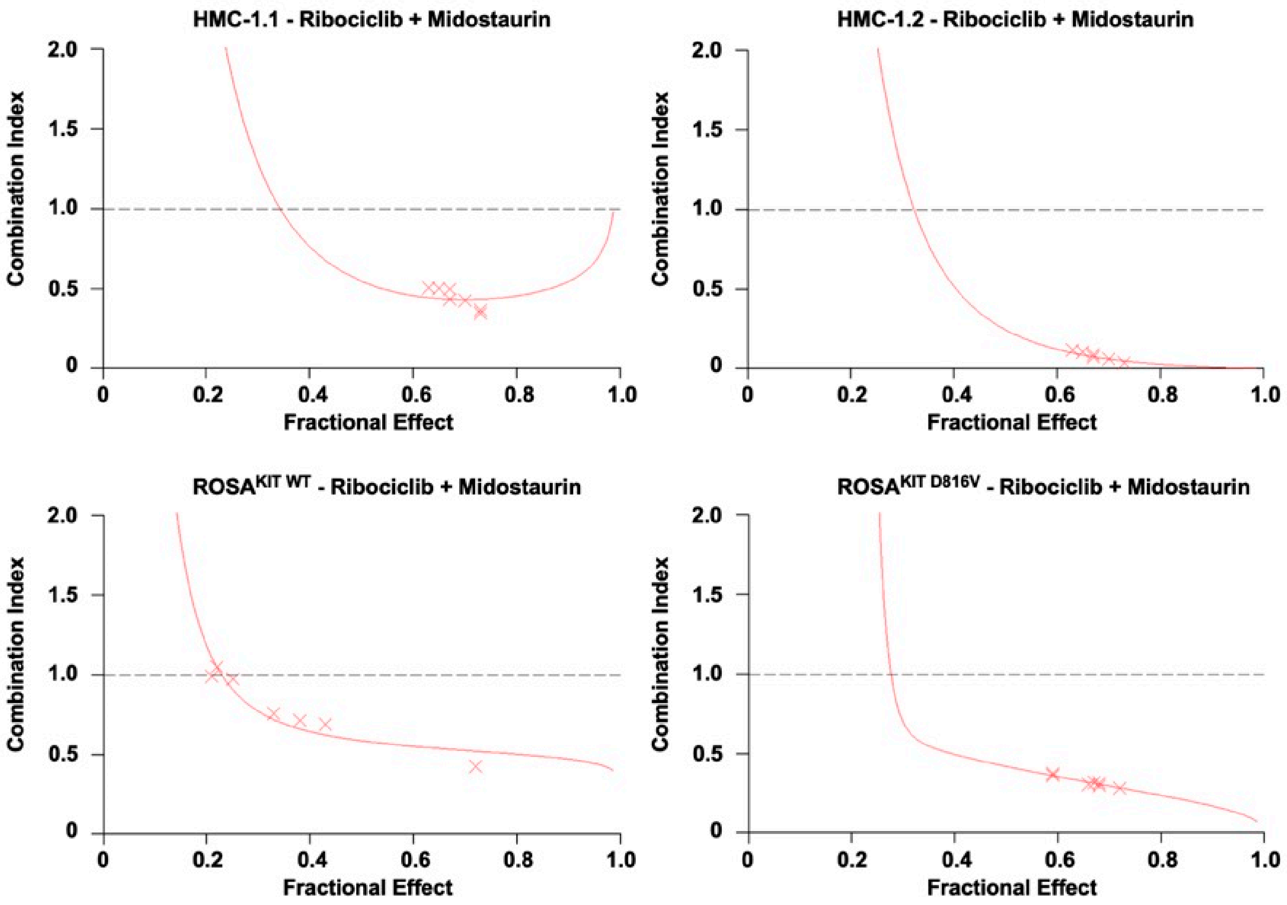

Schneeweiss-Gleixner et al., Supplemental Figure S4B

Synergistic effects of ribociclib and midostaurin applied in combination in MCL-like cell lines as determined by Calcusyn software

HMC-1.1, HMC-1.2, ROSA$^{KIT\ WT}$ and ROSA$^{KIT\ D816V}$ were kept in control medium or in various concentrations of ribocilcib, midostaurin or a combination of both drugs at a fixed ratio at 37°C for 48 hours. Thereafter, cell proliferation was determined by measuring ^{3}H-thymidine uptake as shown in Figure 5B in the main document. The nature of drug interaction (additive versus synergistic) was determined for each experiment by calculating combination index (CI) values using Calcusyn software. The resulting CI values are shown. A CI value of 1 indicates an additive effect, whereas CI values below 1 indicate synergistic drug effects.

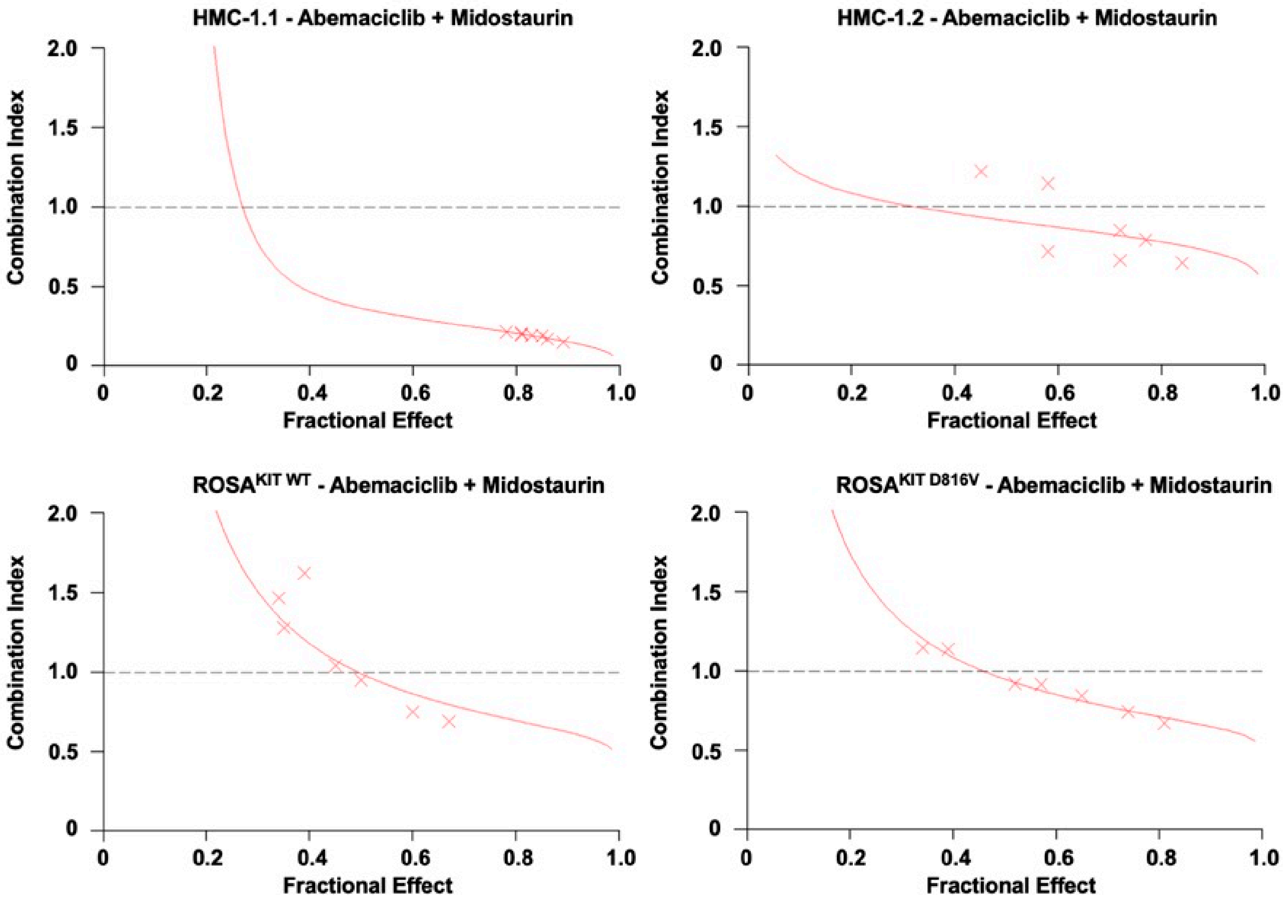

Schneeweiss-Gleixner et al., Supplemental Figure S4C

Synergistic effects of abemaciclib and midostaurin applied in combination in MCL-like cell lines as determined by Calcusyn software

HMC-1.1, HMC-1.2, $ROSA^{KIT\ WT}$ and $ROSA^{KIT\ D816V}$ were kept in control medium or in various concentrations of abemaciclib, midostaurin or a combination of both drugs at a fixed ratio at 37°C for 48 hours. Thereafter, cell proliferation was determined by measuring ^{3}H-thymidine uptake as shown in Figure 5C in the main document. The nature of drug interaction (additive versus synergistic) was determined for each experiment by calculating combination index (CI) values using Calcusyn software. The resulting CI values are shown. A CI value of 1 indicates an additive effect, whereas CI values below 1 indicate synergistic drug effects.

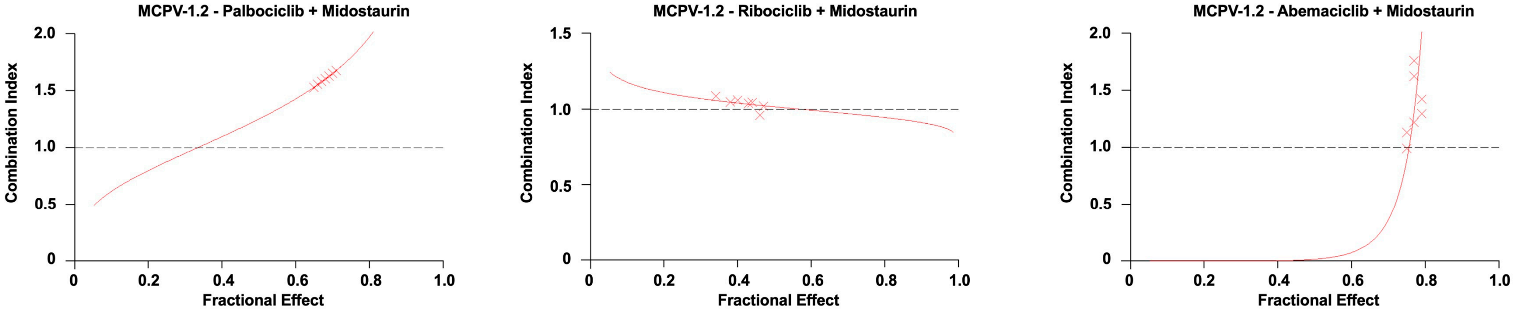

Schneeweiss-Gleixner et al., Supplemental Figure S4D

Synergistic effects of CDK4/CDK6 inhibitors and midostaurin applied in combination in highly resistant MCPV-1 cells as determined by Calcusyn software

MCPV-1.2 cells were kept in control medium or in various concentrations of palbociclib, ribociclib, abemaciclib and midostaurin alone or as drug combination with midostaurin (as indicated) at a fixed ratio at 37°C for 48 hours. Thereafter, cell proliferation was determined by measuring ^{3}H-thymidine uptake as shown in Figure 5F in the main document. The nature of drug interaction (additive versus synergistic) was determined for each experiment by calculating combination index (CI) values using Calcusyn software. The resulting CI values are shown. A CI value of 1 indicates an additive effect, whereas CI values below 1 indicate synergistic drug effects.

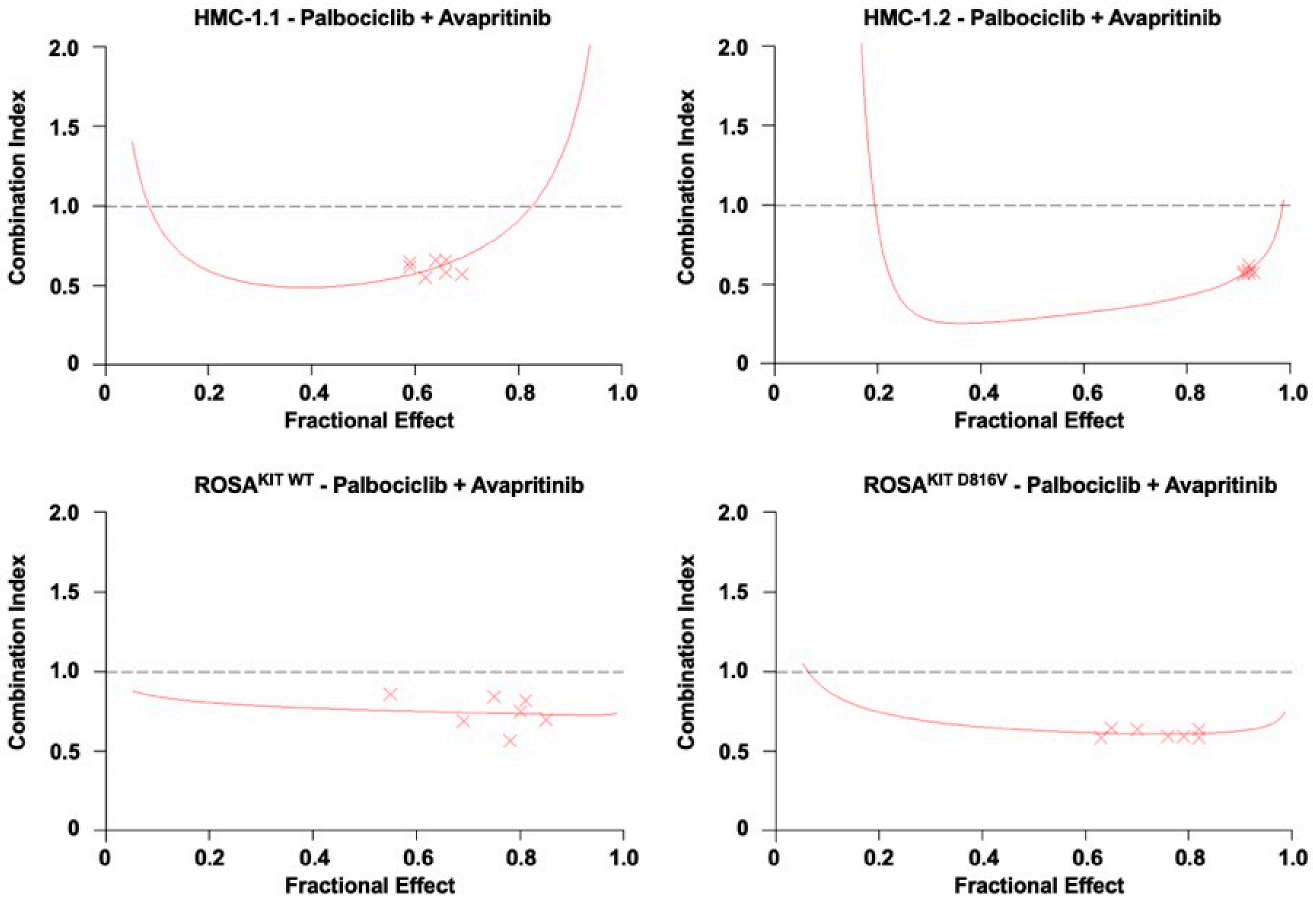

Schneeweiss-Gleixner et al., Supplemental Figure S5A

Synergistic effects of palbociclib and avapritinib applied in combination in MCL-like cell lines as determined by Calcusyn software

HMC-1.1, HMC-1.2, ROSA$^{KIT\ WT}$ and ROSA$^{KIT\ D816V}$ were kept in control medium or in various concentrations of palbociclib, avapritinib or a combination of both drugs at a fixed ratio at 37°C for 48 hours. Thereafter, cell proliferation was determined by measuring ^{3}H-thymidine uptake as shown in Figure 6A in the main document. The nature of drug interaction (additive versus synergistic) was determined for each experiment by calculating combination index (CI) values using Calcusyn software. The resulting CI values are shown. A CI value of 1 indicates an additive effect, whereas CI values below 1 indicate synergistic drug effects.

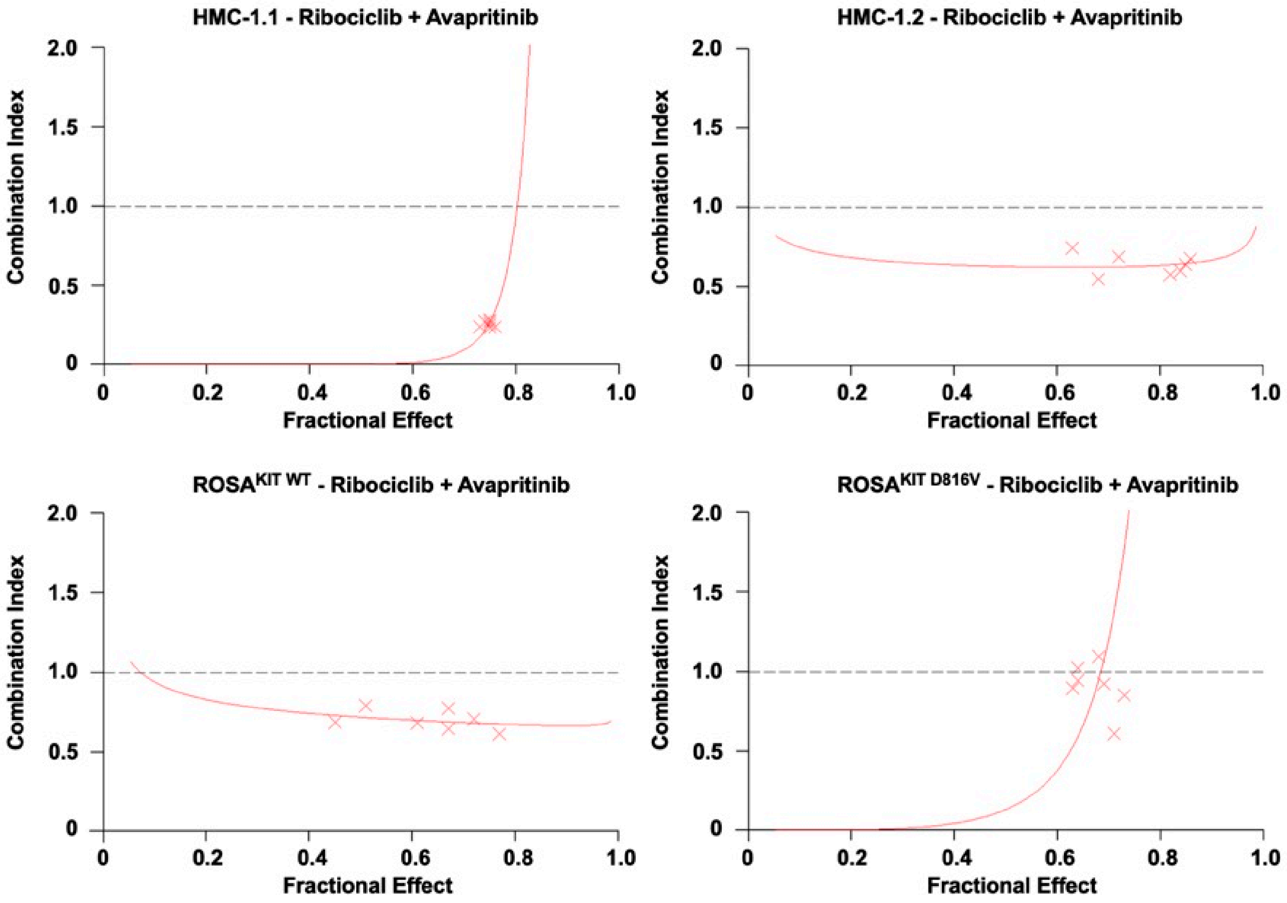

Schneeweiss-Gleixner et al., Supplemental Figure S5B

Synergistic effects of ribociclib and avapritinib applied in combination in MCL-like cell lines as determined by Calcusyn software

HMC-1.1, HMC-1.2, ROSA$^{KIT\ WT}$ and ROSA$^{KIT\ D816V}$ were kept in control medium or in various concentrations of ribociclib, avapritinib or a combination of both drugs at a fixed ratio at 37°C for 48 hours. Thereafter, cell proliferation was determined by measuring ^{3}H-thymidine uptake as shown in Figure 6B in the main document. The nature of drug interaction (additive versus synergistic) was determined for each experiment by calculating combination index (CI) values using Calcusyn software. The resulting CI values are shown. A CI value of 1 indicates an additive effect, whereas CI values below 1 indicate synergistic drug effects.

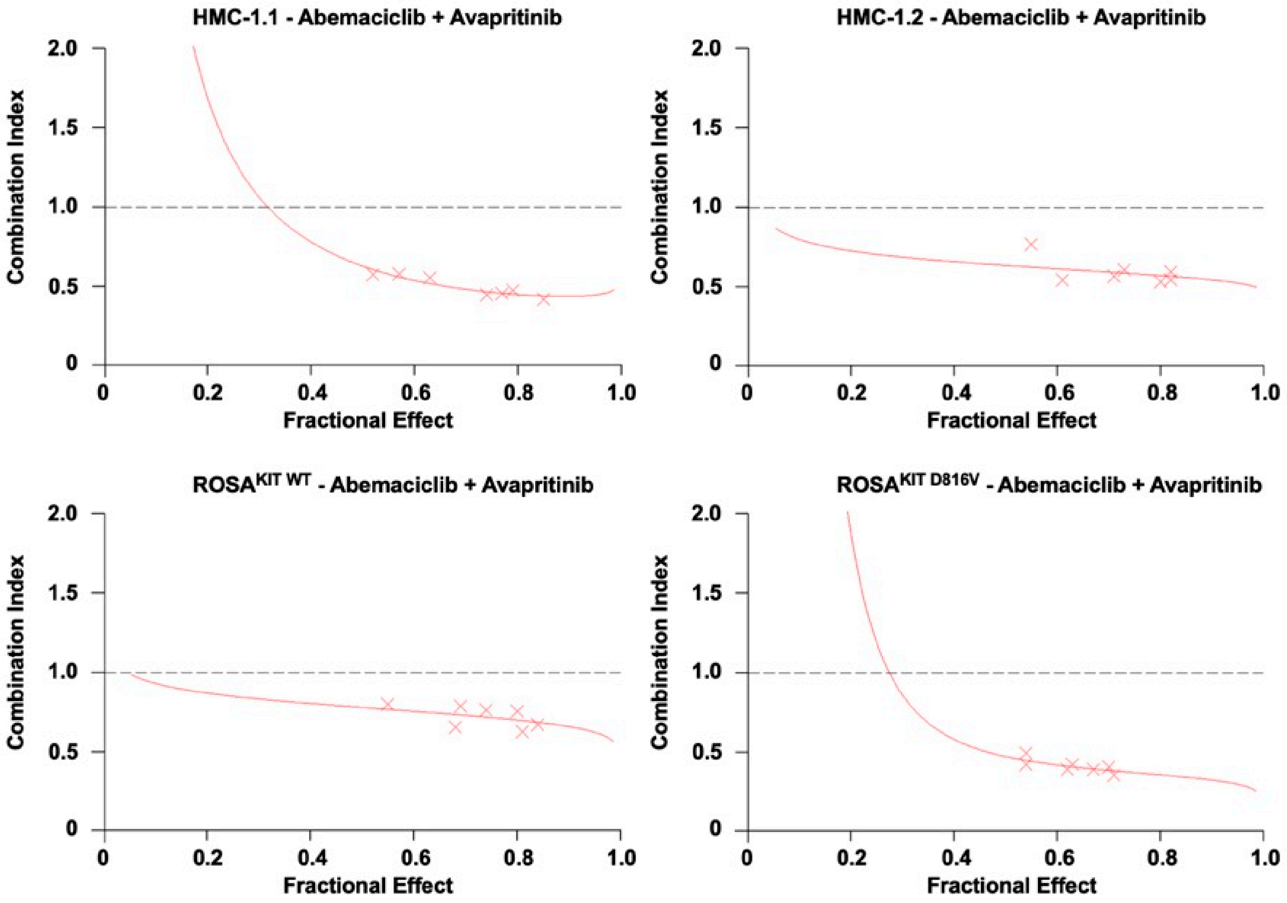

Schneeweiss-Gleixner et al., Supplemental Figure S5C

Synergistic effects of abemaciclib and avapritinib applied in combination in MCL-like cell lines as determined by Calcusyn software

HMC-1.1, HMC-1.2, $ROSA^{KIT\ WT}$ and $ROSA^{KIT\ D816V}$ were kept in control medium or in various concentrations of abemaciclib, avapritinib or a combination of both drugs at a fixed ratio at 37°C for 48 hours. Thereafter, cell proliferation was determined by measuring ^{3}H-thymidine uptake as shown in Figure 6C in the main document. The nature of drug interaction (additive versus synergistic) was determined for each experiment by calculating combination index (CI) values using Calcusyn software. The resulting CI values are shown. A CI value of 1 indicates an additive effect, whereas CI values below 1 indicate synergistic drug effects.

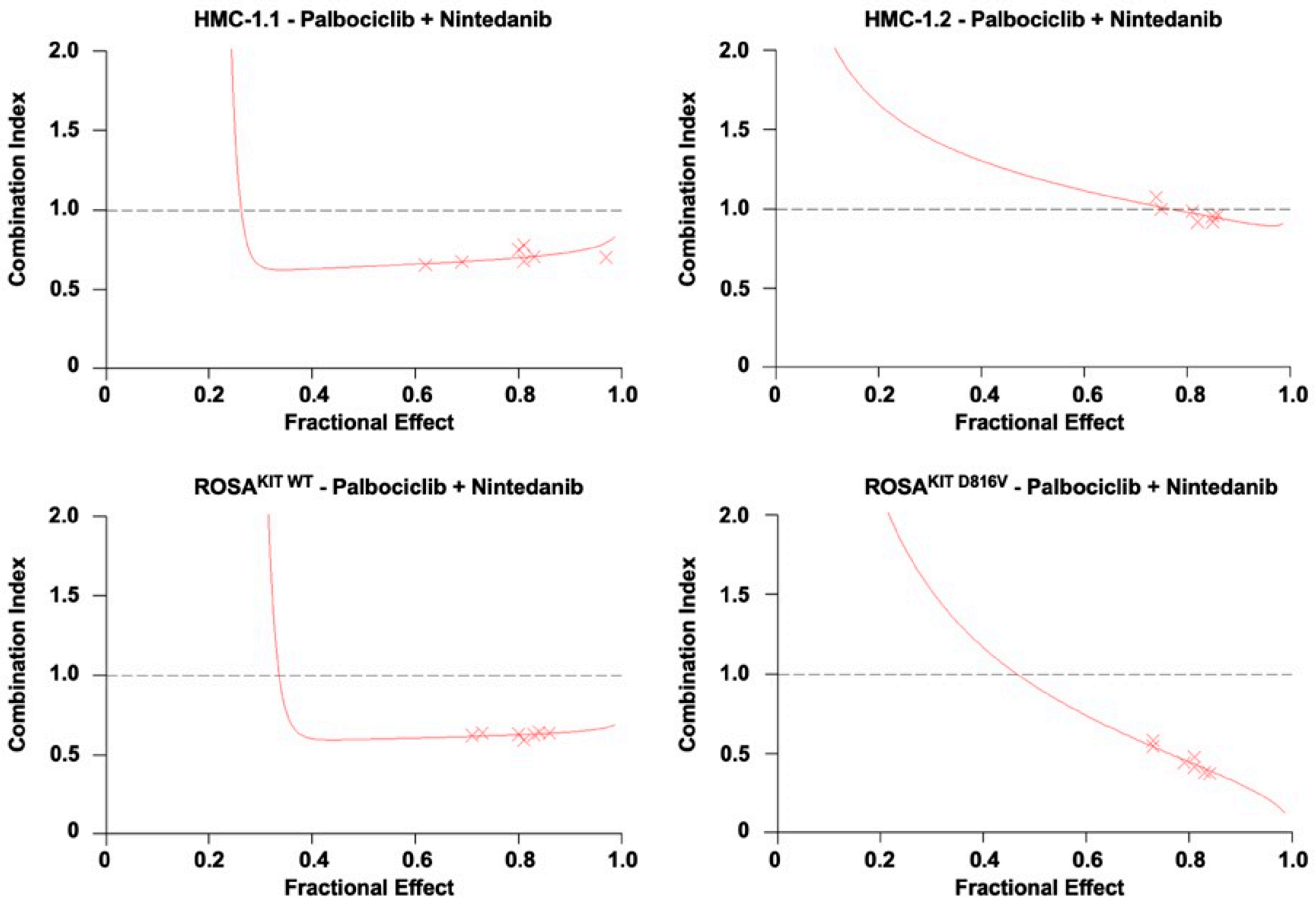

Schneeweiss-Gleixner et al., Supplemental Figure S5D

Synergistic effects of palbociclib and nintedanib applied in combination in MCL-like cell lines as determined by Calcusyn software

HMC-1.1, HMC-1.2, ROSA$^{KIT\ WT}$ and ROSA$^{KIT\ D816V}$ were kept in control medium or in various concentrations of palbociclib, nintedanib or a combination of both drugs at a fixed ratio at 37°C for 48 hours. Thereafter, cell proliferation was determined by measuring ^{3}H-thymidine uptake as shown in Figure 6D in the main document. The nature of drug interaction (additive versus synergistic) was determined for each experiment by calculating combination index (CI) values using Calcusyn software. The resulting CI values are shown. A CI value of 1 indicates an additive effect, whereas CI values below 1 indicate synergistic drug effects.

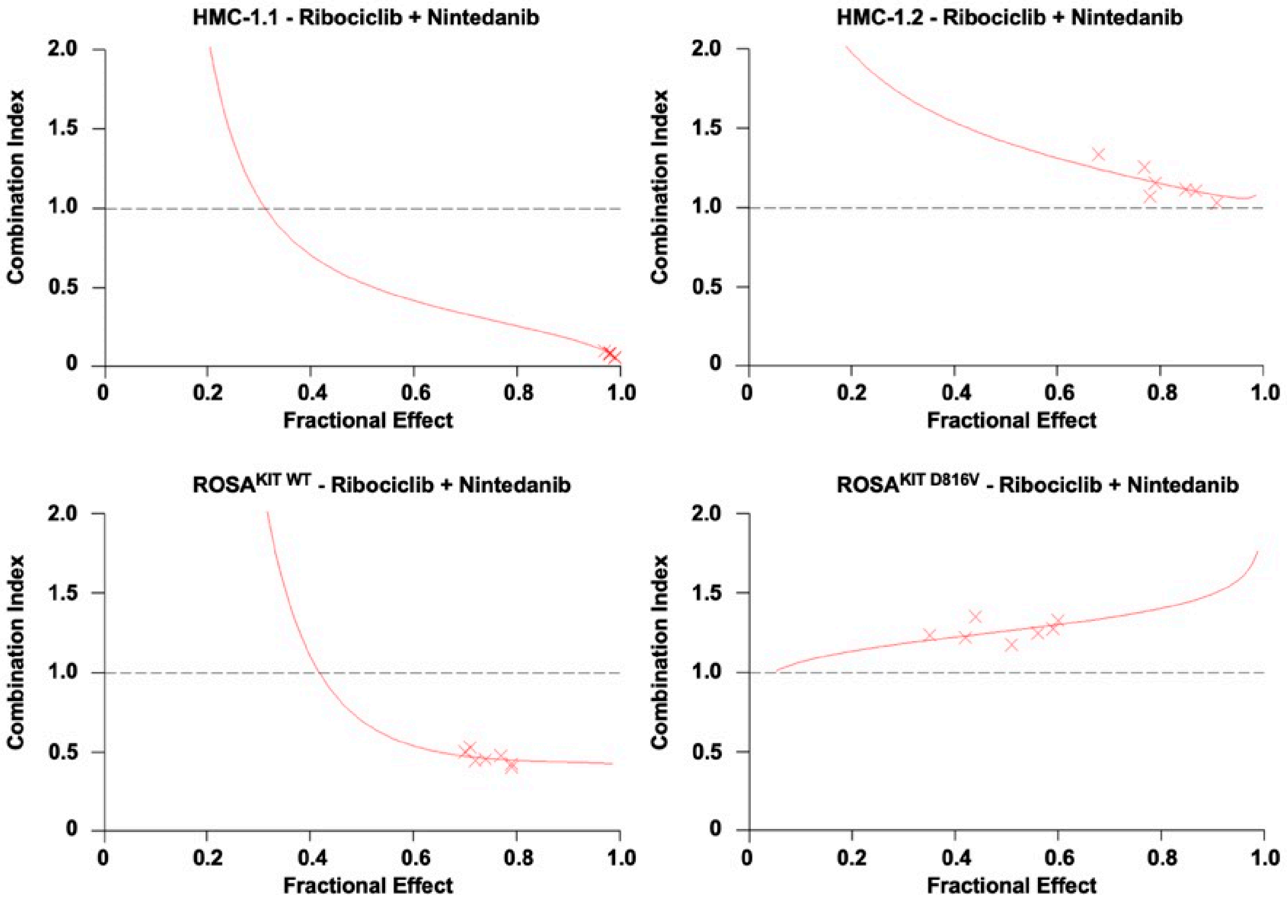

Schneeweiss-Gleixner et al., Supplemental Figure S5E

Synergistic effects of ribociclib and nintedanib applied in combination in MCL-like cell lines as determined by Calcusyn software

HMC-1.1, HMC-1.2, ROSA$^{KIT\ WT}$ and ROSA$^{KIT\ D816V}$ were kept in control medium or in various concentrations of ribociclib, nintedanib or a combination of both drugs at a fixed ratio at 37°C for 48 hours. Thereafter, cell proliferation was determined by measuring ^{3}H-thymidine uptake as shown in Figure 6E in the main document. The nature of drug interaction (additive versus synergistic) was determined for each experiment by calculating combination index (CI) values using Calcusyn software. The resulting CI values are shown. A CI value of 1 indicates an additive effect, whereas CI values below 1 indicate synergistic drug effects.

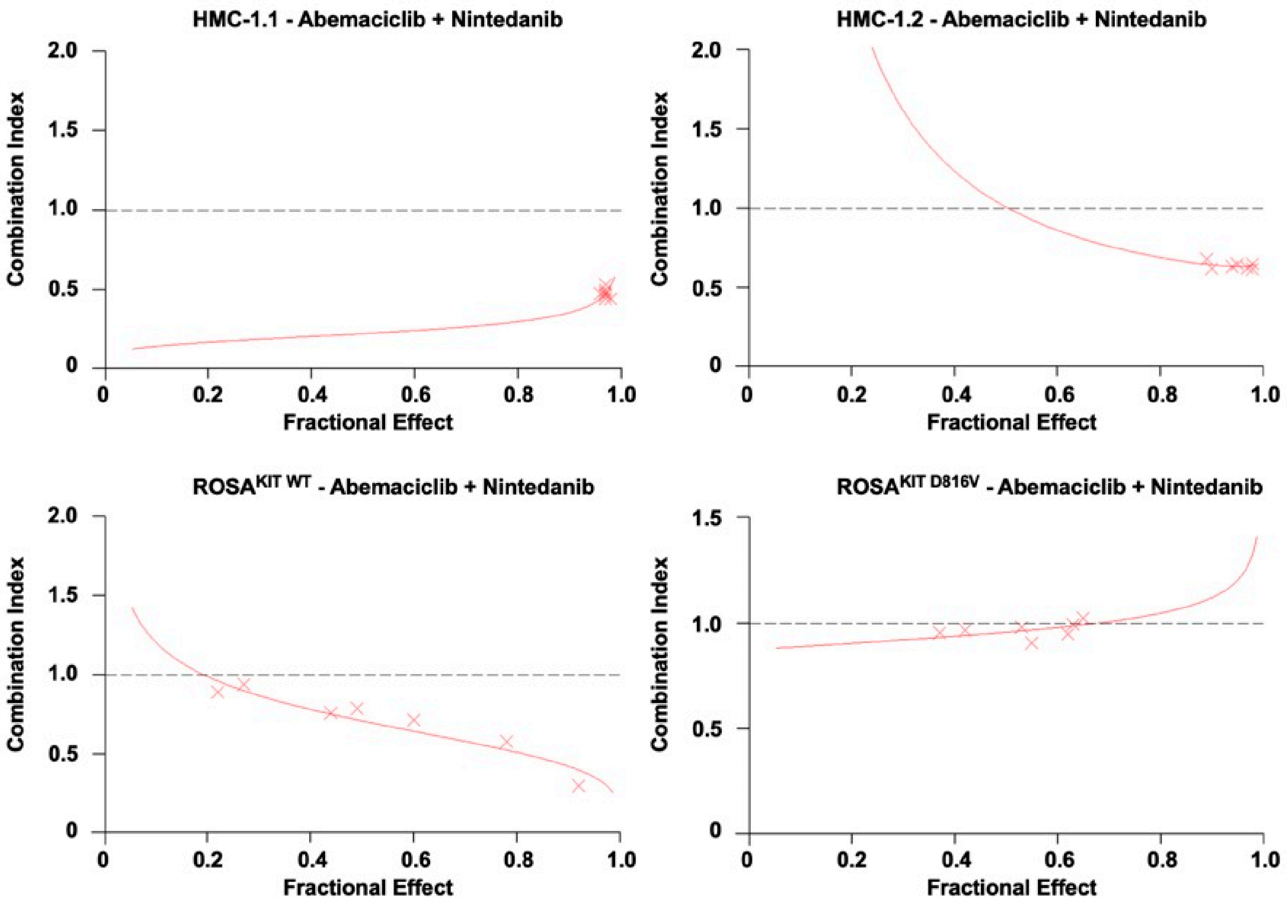

Schneeweiss-Gleixner et al., Supplemental Figure S5F

Synergistic effects of abemaciclib and nintedanib applied in combination in MCL-like cell lines as determined by Calcusyn software

HMC-1.1, HMC-1.2, ROSA$^{KIT\ WT}$ and ROSA$^{KIT\ D816V}$ were kept in control medium or in various concentrations of abemaciclib, nintedanib or a combination of both drugs at a fixed ratio at 37°C for 48 hours. Thereafter, cell proliferation was determined by measuring ^{3}H-thymidine uptake as shown in Figure 6F in the main document. The nature of drug interaction (additive versus synergistic) was determined for each experiment by calculating combination index (CI) values using Calcusyn software. The resulting CI values are shown. A CI value of 1 indicates an additive effect, whereas CI values below 1 indicate synergistic drug effects.

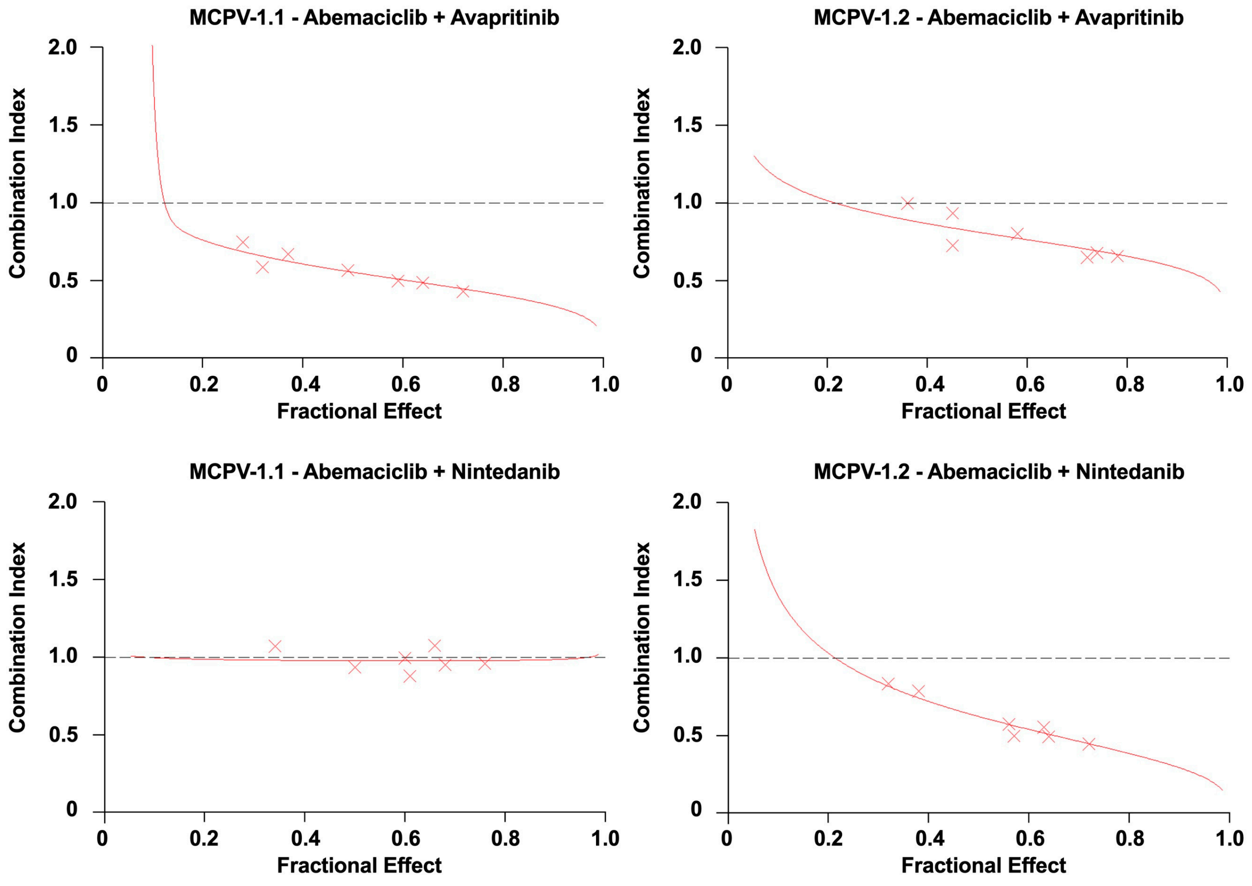

Schneeweiss-Gleixner et al., Supplemental Figure S5G

Synergistic effects of abemaciclib and avapritinib applied in combination in highly resistant MCPV-1 cells as determined by Calcusyn software

MCPV-1.1 and MCPV-1.2 cells were incubated with control medium (0 µM) or medium containing various concentrations of abemaciclib alone or in combination with avapritinib (upper row) or nintedanib (lower row) at a fixed ratio at 37°C for 48 hours. Thereafter, cell proliferation was determined by measuring ^{3}H-thymidine uptake as shown in Figure 6G in the main document. The nature of drug interaction (additive versus synergistic) was determined for each experiment by calculating combination index (CI) values using Calcusyn software. The resulting CI values are shown. A CI value of 1 indicates an additive effect, whereas CI values below 1 indicate synergistic drug effects.

V. Additional Information: original western blots and densitometric analysis

Original blots corresponding to Figure 1A

CDK4

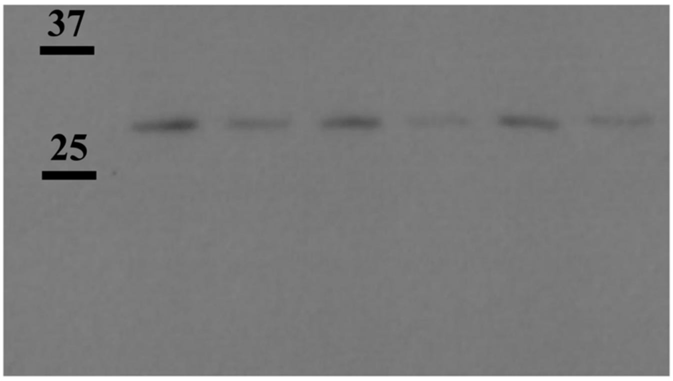

CDK6

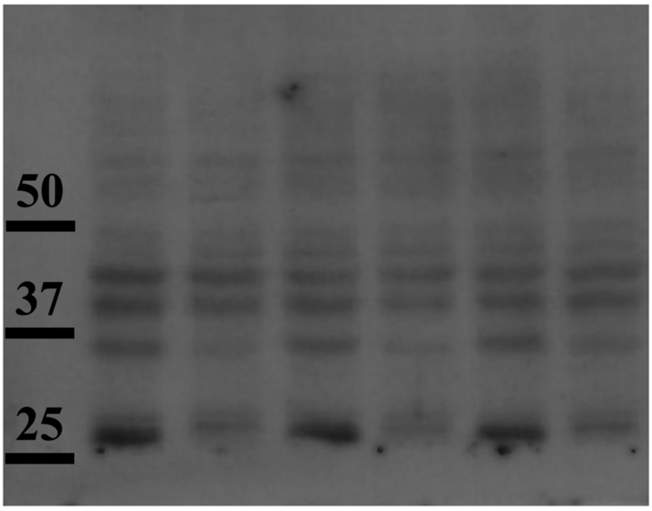

β-Tubulin

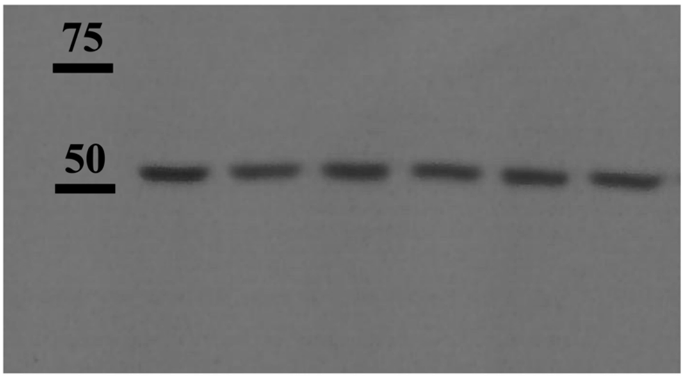

Original blots corresponding to Figure 2A (HMC1.1)

pRB1

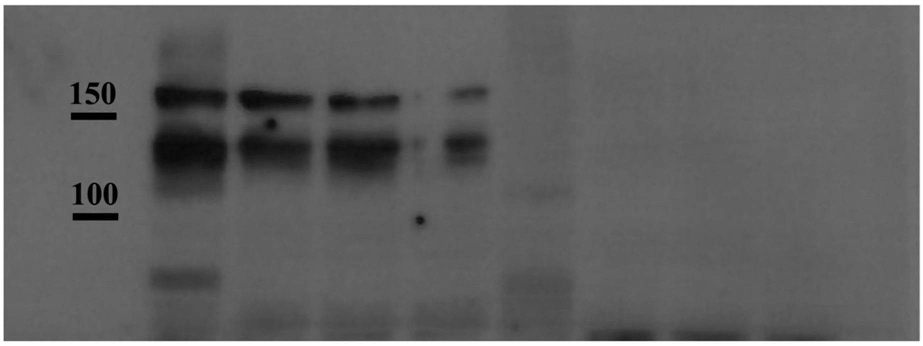

RB1

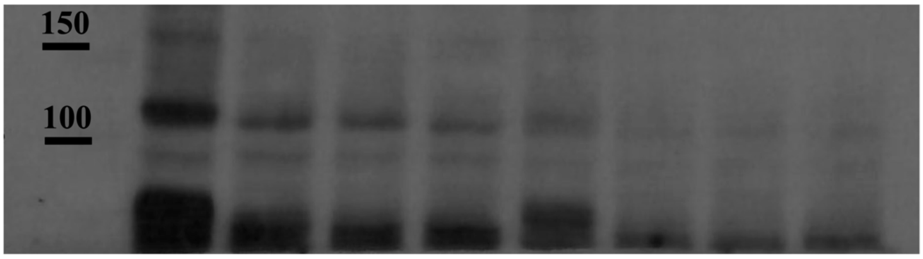

Actin

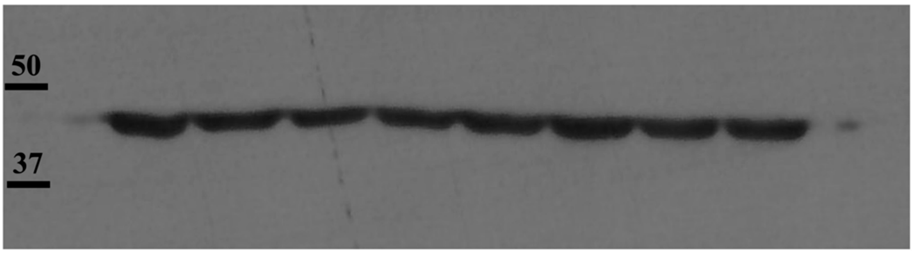

<u>Original blots corresponding to Figure 2A (HMC1.2)</u>

pRB1

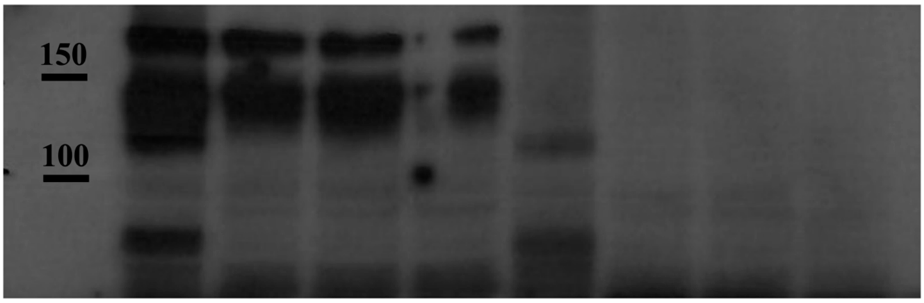

RB1

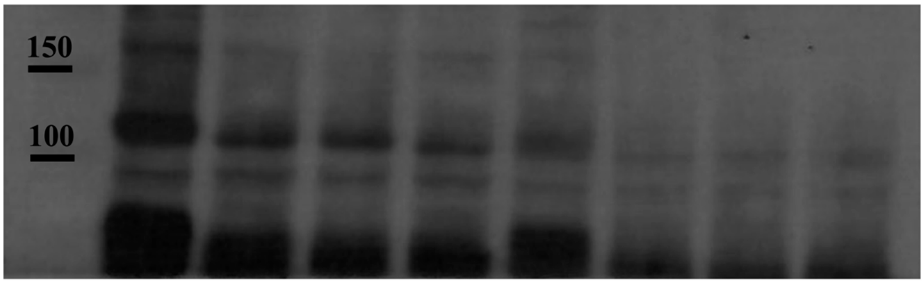

Actin

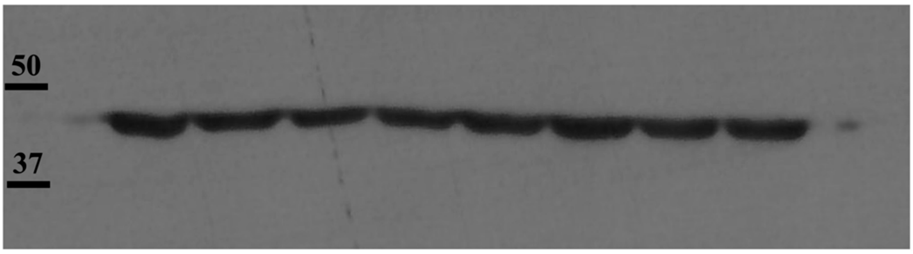

Original blots corresponding to Figure 2B

pRB1

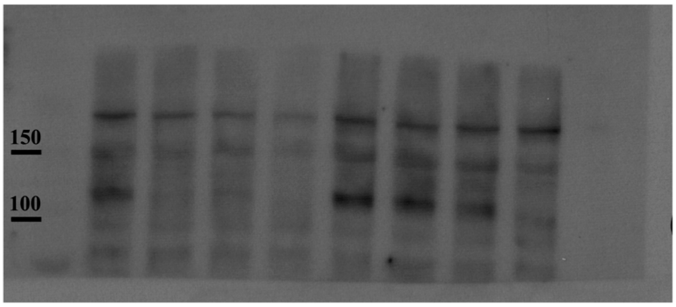

RB1

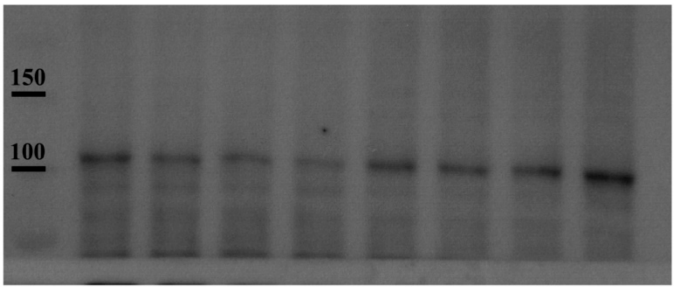

Actin

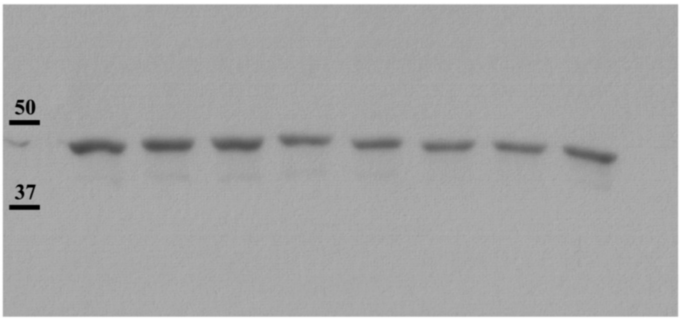

Original blots corresponding to Figure 5E

Cleaved Caspase-3

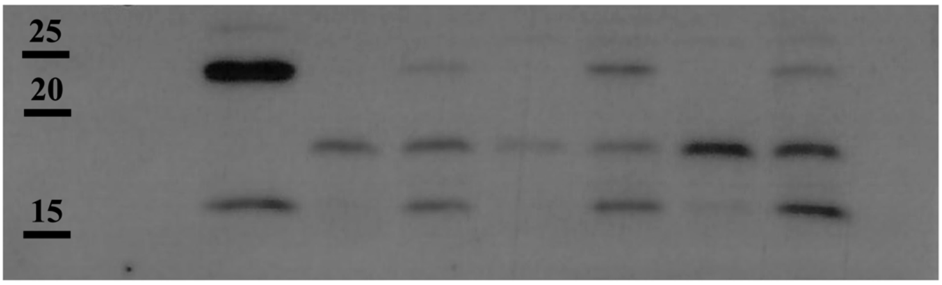

β-Tubulin

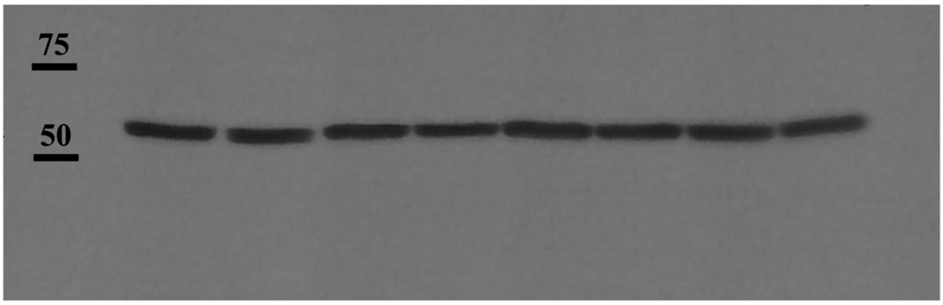

	CDK6		CDK4		Actin
	Signal	Ratio	Signal	Ratio	Signal
**HMC-1.2 NTC + Doxycycline**	24429317	0.99	19320075	0.79	24576903
**HMC-1.2 shCDK4/6 + Doxycycline**	11335468	0.63	10448125	0.58	18111832
**HMC-1.2 NTC + Doxycycline**	18237539	0.75	17041075	0.70	24478782
**HMC-1.2 shCDK4/6 + Doxycycline**	7977246	0.41	7055953	0.36	19395711
**HMC-1.2 NTC + Doxycycline**	18676004	0.79	15612782	0.66	23606489
**HMC-1.2 shCDK4/6 + Doxycycline**	12589418	0.60	8368882	0.40	20984125

Densitometric analysis of corresponding western Blot (Figure 1A): Ratio relative to actin for corresponding cell line. Abbreviations: CDK, cyclin-dependent kinase; RB1, retinoblastoma protein; NTC, targeting control shRNAs; shCDK4/6, shRNAs directed against CDK4 and CDK6.

HMC-1.1	pRB1			RB1			Actin
	Signal	Ratio to actin	Density (% of control)	Signal	Ratio to actin	Density (% of control)	Signal
**Control**	38500723	1.19	100	39836530	1.23	100	32302489
**Palbociclib 1µM**	9690045	0.35	29	21274530	0.76	62	28075782
**Ribociclib 1µM**	18817602	0.81	68	16316075	0.70	57	23353004
**Abemaciclib 1µM**	4003175	0.16	13	13901903	0.57	46	24478004

HMC-1.2	pRB1			RB1			Actin
	Signal	Ratio to actin	Density (% of control)	Signal	Ratio to actin	Density (% of control)	Signal
**Control**	40918602	1.40	100	38853530	1.33	100	29290418
**Palbociclib 1µM**	13599217	0.39	28	17925874	0.52	39	34432539
**Ribociclib 1µM**	18239024	0.69	49	14636903	0.56	42	26319296
**Abemaciclib 1µM**	14181711	0.51	36	16420066	0.59	44	27977246

Densitometric analysis of corresponding western Blot (Figure 2A): Ratio relative to actin for corresponding condition; density was calculated as percent (%) of control (the ratio pRB1/actin and RB1/actin respectively for cells kept under control conditions represent 100%). Abbreviations: CDK, cyclin-dependent kinase; RB1, retinoblastoma protein; pRB1, phosphorylated RB1.

ROSA$^{KIT\ WT}$	pRB1			RB1			Actin
	Signal	Ratio to actin	Density (% of control)	Signal	Ratio to actin	Density (% of control)	Signal
**Control**	57060116	0.97	100	50873359	0.86	100	59075238
**Palbociclib 1µM**	31284480	0.61	63	38501945	0.75	87	51255681
**Ribociclib 1µM**	30702602	0.62	64	32441016	0.66	77	49256681
**Abemaciclib 1µM**	18604480	0.52	54	26343066	0.74	86	35531388

ROSA$^{KIT\ D816V}$	pRB1			RB1			Actin
	Signal	Ratio to actin	Density (% of control)	Signal	Ratio to actin	Density (% of control)	Signal
**Control**	58398016	1.65	100	38669309	1.09	100	35435267
**Palbociclib 1µM**	61268551	1.82	110	36104430	1.07	98	33730631
**Ribociclib 1µM**	52477309	1.64	99	42870894	1.34	123	32044702
**Abemaciclib 1µM**	31412752	0.70	42	62259238	1.39	128	44931794

Densitometric analysis of corresponding western Blot (Figure 2B): Ratio relative to actin for corresponding condition; density was calculated as percent (%) of control (the ratio pRB1/actin and RB1/actin respectively for cells kept under control conditions represent 100%). Abbreviations: CDK, cyclin-dependent kinase; RB1, retinoblastoma protein; pRB1, phosphorylated RB1.

	**Cleaved Caspase-3**		**Actin**
	Signal	Ratio	Signal
**Control HMC-1.1**	122314	0.00	33705368
**Palbociclib + Midostaurin HMC-1.1**	18895974	0.64	29546832
**Control HMC-1.2**	1138205	0.04	30866418
**Palbociclib + Midostaurin HMC-1.2**	8911761	0.33	26867296
**Control ROSA$^{KIT\ WT}$**	275728	0.01	34735246
**Palbociclib + Midostaurin ROSA$^{KIT\ WT}$**	15579660	0.51	30577711
**Control ROSA$^{KIT\ D816V}$**	4588397	0.13	34187368
**Palbociclib + Midostaurin ROSA$^{KIT\ D816V}$**	26267660	0.92	28655368

Densitometric analysis of corresponding western Blot (Figure 5E): Ratio relative to ß-tubulin for corresponding condition.
